# Improved Salinity Tolerance of Rice Through Cell Type-Specific Expression of *AtHKT1;1*

**DOI:** 10.1371/journal.pone.0012571

**Published:** 2010-09-03

**Authors:** Darren Plett, Gehan Safwat, Matthew Gilliham, Inge Skrumsager Møller, Stuart Roy, Neil Shirley, Andrew Jacobs, Alexander Johnson, Mark Tester

**Affiliations:** 1 Australian Centre for Plant Functional Genomics, University of Adelaide, Glen Osmond, South Australia, Australia; 2 School of Agriculture, Food and Wine, Waite Research Institute, University of Adelaide, Glen Osmond, South Australia, Australia; Iwate University, Japan

## Abstract

Previously, cell type-specific expression of *AtHKT1;1*, a sodium transporter, improved sodium (Na^+^) exclusion and salinity tolerance in Arabidopsis. In the current work, *AtHKT1;1*, was expressed specifically in the root cortical and epidermal cells of an Arabidopsis GAL4-GFP enhancer trap line. These transgenic plants were found to have significantly improved Na^+^ exclusion under conditions of salinity stress. The feasibility of a similar biotechnological approach in crop plants was explored using a GAL4-GFP enhancer trap rice line to drive expression of *AtHKT1;1* specifically in the root cortex. Compared with the background GAL4-GFP line, the rice plants expressing *AtHKT1;1* had a higher fresh weight under salinity stress, which was related to a lower concentration of Na^+^ in the shoots. The root-to-shoot transport of ^22^Na^+^ was also decreased and was correlated with an upregulation of *OsHKT1;5*, the native transporter responsible for Na^+^ retrieval from the transpiration stream. Interestingly, in the transgenic Arabidopsis plants overexpressing *AtHKT1;1* in the cortex and epidermis, the native *AtHKT1;1* gene responsible for Na^+^ retrieval from the transpiration stream, was also upregulated. Extra Na^+^ retrieved from the xylem was stored in the outer root cells and was correlated with a significant increase in expression of the vacuolar pyrophosphatases (in Arabidopsis and rice) the activity of which would be necessary to move the additional stored Na^+^ into the vacuoles of these cells. This work presents an important step in the development of abiotic stress tolerance in crop plants via targeted changes in mineral transport.

## Introduction

Salinity stress affects crop production worldwide, particularly by limiting plant growth and reducing potential yield. Plants have three mechanisms for tolerating soil salinity, including tolerance of the osmotic effects of salt; a tolerance in leaf tissues of the negative effects of sodium ions (Na^+^) on cellular function through compartmentalisation of Na^+^ in specific tissues, cells, or cellular organelles; and exclusion of Na^+^ from the sensitive shoot tissue [1]. Of these mechanisms, Na^+^ exclusion from the shoot is best understood and is, therefore, the current best candidate for a targeted genetic modification approach to improving salinity tolerance.

The *HKT* gene family encodes one of the most widely studied groups of Na^+^ transporters in plants. Current understanding indicates that *AtHKT1;1* from Arabidopsis is expressed primarily in the stele and has a role in the retrieval of Na^+^ from the transpiration stream [2], [3]. Reduced activity of this transporter leads to increased transfer of Na^+^ from the root to the shoot, leading to a higher shoot Na^+^ concentration and a decrease in salinity tolerance [4]. More recently, it was demonstrated that OsHKT1;5 in rice [5] and the *HKT1;5* gene from wheat [6] are crucial in maintaining low shoot Na^+^ concentrations via a similar mechanism of Na+ retrieval from the transpiration stream into cells within the stele. A second HKT gene that is involved in sodium exclusion in cereal species is *HKT1;4*. The encoded transporter appears to function in combination with HKT1;5, by retrieving Na^+^ from the shoot transpiration stream and sequestering Na^+^ in the sheath tissue [7]. The endogenous function of the family of HKT transporters and their role in Na^+^ transport and provision of salinity tolerance to plants has been reviewed recently [8].

An obvious strategy for improving the salinity tolerance of plants is to increase the expression of *HKT* genes. Constitutive overexpression of plasma membrane Na^+^ transporters, however, has been unsuccessful in producing salinity tolerant plants [9]. It was hypothesized that such overexpression increases Na^+^ fluxes into all cells of the plant, this being, on balance, counter-productive to the reduction of Na^+^ transport to the shoot [9], [10]. In contrast, it was shown that overexpression of *AtHKT1;1* specifically within the root xylem parenchyma cells of Arabidopsis leads to improved Na^+^ exclusion and salinity tolerance [9]. Interestingly, the excess Na^+^ in the root resulting from the improved Na^+^ exclusion from the shoot was stored within the cortical cells of the root [9].

The aim of experiments described in the current work was to determine if overexpression of *AtHKT1;1* specifically within the outer cells (epidermis and cortex) of the root would result in opposite effects to overexpression in the stele, or whether a similar improvement of Na^+^ exclusion and salinity tolerance of Arabidopsis and rice would be observed. A secondary goal was to determine if expression of *AtHKT1;1* in the epidermal and cortical cell layers of the root is the reason for the deleterious effects observed when *AtHKT1;1* is constitutively overexpressed, as described previously [9].

In both species, we found that transgenic plants overexpressing *AtHKT1;1* in mature root cortex had greater shoot Na^+^ exclusion and thus increased salinity tolerance.

## Results

### Expression of *AtHKT1;1* in the root epidermis and cortex of Arabidopsis decreases Na^+^ accumulation in the shoot

**Figure 1:**
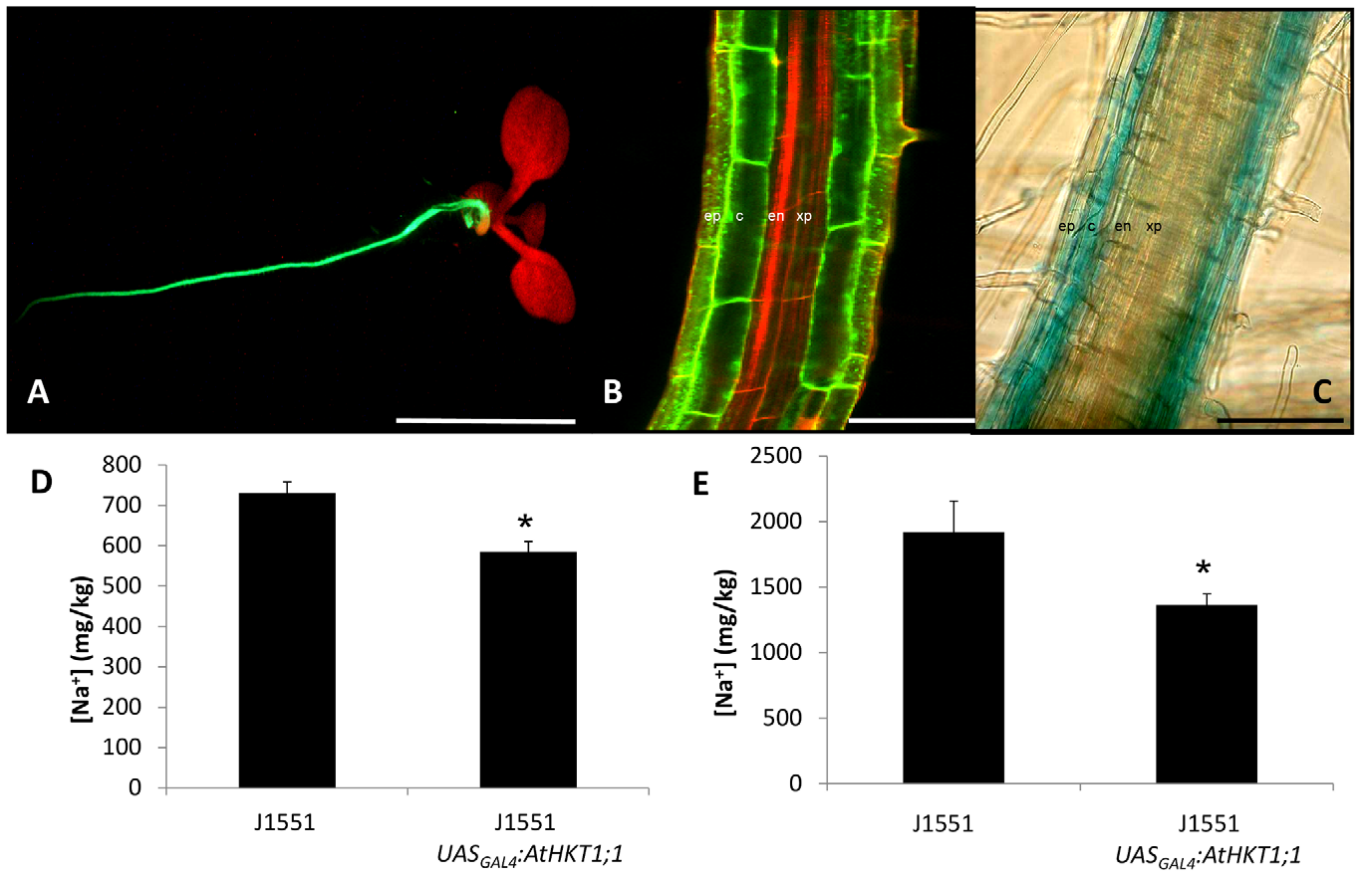
Arabidopsis plants expressing *AtHKT1;1* within the root epidermal and cortical cells. (A) Representative fluorescence stereomicroscope and (B) confocal laser microscope images of J1551 showing GFP fluorescence specifically within the root epidermal and cortical cells. Tissue was stained with propidium iodide. GFP and propidium iodide images were captured separately and overlaid to create the composite image. Tissues labelled include the epidermis (ep), cortex (c), endodermis (en) and xylem parenchyma (xp). (C) Transactivation of *uidA* in J1551 produces GUS staining specifically within the root epidermal and cortical cells [Scale bars  = 10 mm in (A), 75 µm in (B) and 75 µm in (C)]. (D) Concentration of Na^+^ within the leaves of T1 J1551 and J1551 *UAS_GAL4_:AtHKT1;1* plants grown on soil and watered with nutrient solution containing 2 mM NaCl (n = 12 for background and 45 independent events for *AtHKT1;1*, error bars represent SEM). (E) Accumulation of Na^+^ within the leaves of T2 J1551 and J1551 *UAS_GAL4_:AtHKT1;1* plants grown on soil and watered with nutrient solution containing 5 mM NaCl (n = 3 for background and 20 for AtHKT1;1 lines for each of 4 independent events, presented as a grand average, error bars represent SEM). Statistical significance from the J1551 line was determined using Student's t-test, *P<0.05.

A homozygous single-insert GAL4-GFP enhancer trap line (J1551) with cell type-specific *mGFP –ER* expression in the epidermal and cortical cell layers of the root (Figures 1A and 1B) was isolated from a library described previously [9], [11]. Epidermal GFP fluorescence appeared slightly less intense than that found in the cortex. To confirm that a gene-of-interest could be transactivated specifically in the same cell-types as GAL4-GFP, the *uidA* gene was fused to a second UAS_GAL4_ element and transformed into J1551. β-glucuronidase activity was detected in the same cell-types as the original GFP fluorescence pattern (Figure 1C) and both GFP and GUS expression was unaffected by NaCl treatment. Therefore, *AtHKT1;1* was transactivated in the J1551 background (J1551 *UAS_GAL4_:AtHKT1;1*) to express *AtHKT1;1* specifically in the mature root epidermal and cortical cells.

Primary Arabidopsis J1551 *UAS_GAL4_:AtHKT1;1* transformants grown in soil and treated with a low concentration of NaCl (2 mM) showed 20% lower leaf Na (Figure 1D) and significantly higher K, P and Zn (averaged over all independent T1 lines produced) than J1551 (Table S1). Four independent lines were selected to test heritability in the T2 generation. The four lines were grown in soil and were sprayed with Basta to remove null segregants. Each of the four independent lines retained 16 to 37% lower shoot Na content (treated with 5 mM NaCl) than J1551 in the T2 generation (Figure 1E, Table S2) and maintained higher K (Table S2).

### Expression of *AtHKT1;1* in the root cortex of rice increases salinity tolerance and decreases shoot Na^+^ accumulation

**Figure 2:**
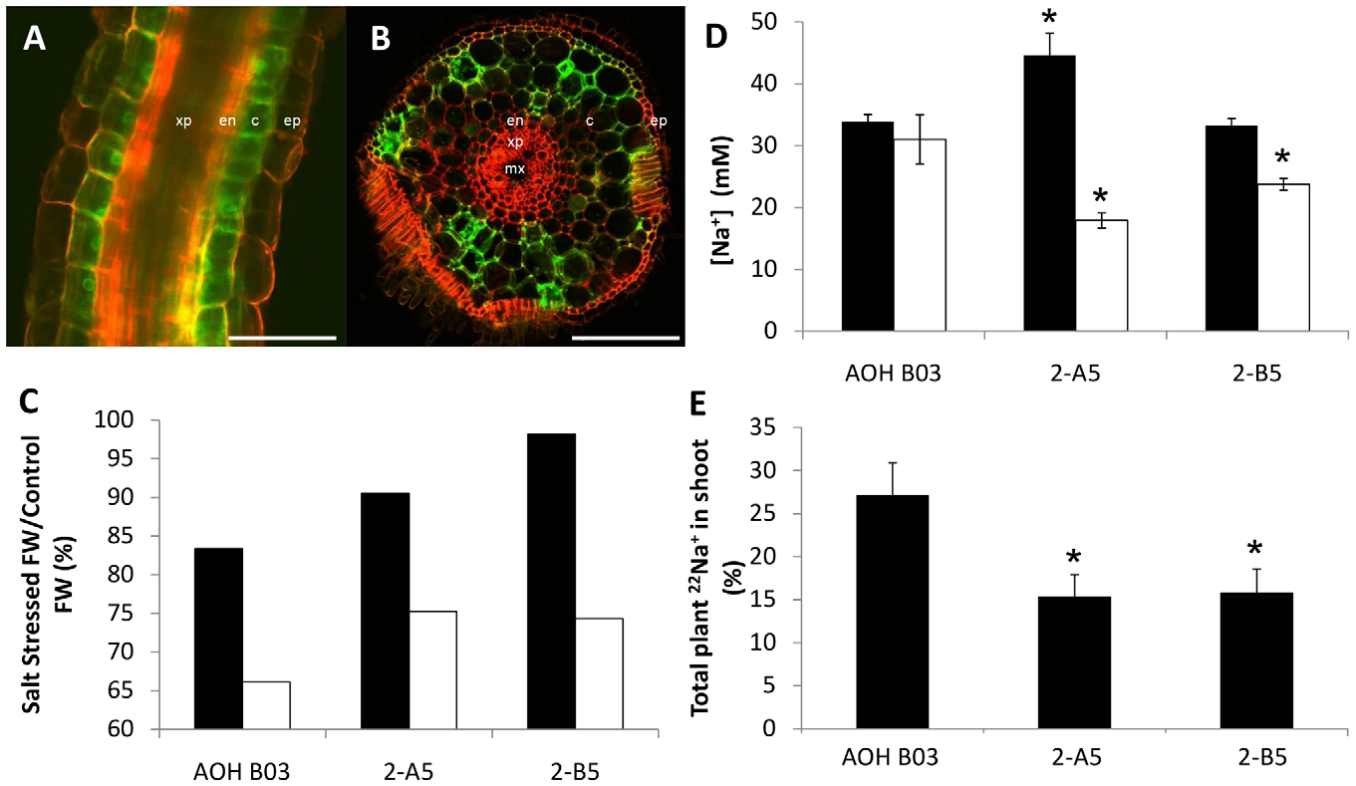
Rice plants expressing *AtHKT1;1* within the root epidermal and cortical cells. (A + B) Representative confocal laser microscope images of line AOH B03 showing GFP fluorescence specifically within the root epidermis and cortical cells [Scale bars  = 100 µm in (A) and 100 µm in (B)]. Tissue was stained with propidium iodide. GFP and propidium iodide images were captured separately and overlaid to create the composite image. Tissues labelled include the epidermis (ep), cortex (c), endodermis (en), xylem parenchyma (xp) and metaxylem (mx). (C) Salinity stress tolerance in AOH B03 and two independently transformed T2 AOH B03 *UAS_GAL4_:AtHKT1;1* rice lines (2-A5 and 2-B5). Plants were stressed for 5 d at 80 mM. Bars represent: ((stress FW/control FW) ×100) in the roots (black bars) and shoots (open bars) (n = 8). (D) Concentration of Na^+^ within the roots (black bars) and shoots (open bars) of AOH B03 and AOH B03 *UAS_GAL4_:AtHKT1;1* lines (2-A5 and 2-B5) stressed for 5 d at 80 mM (n = 8, error bars represent SEM). Statistical significance from the AOH BO3 line for each tissue was determined using Student's t-test, *P<0.05. (E) Total amount of ^22^Na^+^ in the rice plant that is contained within the shoot tissue of the plant (expressed as a percentage) after exposing the roots of intact plants to a solution containing 30 mM Na^+^ for 1 h (n = 8, error bars represent SEM). Statistical significance from the AOH BO3 line was determined using Student's t-test, *P<0.05.

Two lines were selected from the GAL4-GFP enhancer trap library described previously [12], one with GFP fluorescence specifically within the xylem parenchyma of the root (ASG F03) (Figures S1A and S1B), and one with GFP fluorescence specifically within the cortical cell layer of the root (AOH B03) (Figures 2A and 2B). Compared with cortical cells, the GFP fluorescence in epidermal cells was extremely weak (Figure 2A).

The two GAL4 lines were transformed [13], [14] with a *UAS_GAL4_:AtHKT1;1* construct. Two independent T0 lines in each background were selected that accumulated levels of leaf Na^+^ which were average for their respective group of independent T0 lines. T2 lines derived from this material expressing *AtHKT1;1* in the cortical cells of the root (AOH B03 *UAS_GAL4_:AtHKT1;1*) maintained significantly higher fresh weight and lower shoot Na^+^ concentration (and higher K^+^ concentration) when challenged with a Na^+^ stress than AOH B03 (Figure S2). However, the lines expressing *AtHKT1;1* in the xylem parenchyma cells of the root (ASG F03 *UAS_GAL4_:AtHKT1;1*) had similar fresh weights and root Na^+^ concentrations and had higher shoot Na^+^ concentrations (and lower K^+^ concentrations) than ASG F03 (Figure S2). Therefore, the ASG F03 *UAS_GAL4_:AtHKT1;1* lines were eliminated from further analyses.

The two independent T2 AOH B03 *UAS_GAL4_:AtHKT1;1* lines (2-A5 and 2-B5) were better able to maintain growth in saline conditions compared to the parental line, AOH B03 (Figures 2C, S3A and S3B) and had lower shoot Na^+^ and higher shoot K^+^ than the parent, with a higher root Na^+^ concentration in one line (2-A5) (Figures 2D and S3C).

In order to determine which component of Na^+^ transport had been altered by the expression of *AtHKT1;1* in the cortical root cells, resulting in the lowering of shoot Na^+^ concentration in those lines, radiotracer ^22^Na^+^ flux analysis was used to measure the rate of transfer of Na^+^ between various tissues in the plants. This analysis indicated that the root-to-shoot transfer of Na^+^ had been significantly decreased in the two AOH B03 *UAS_GAL4_:AtHKT1;1* lines (15 and 16% versus 27% in control) (Figure 2E). A significant proportion of Na^+^ influx into rice roots is reported to occur through the apoplastic pathway [15], thus excised roots were also analysed for unidirectional influx in an attempt to determine if transport protein-mediated Na^+^ influx had been affected by the expression of *AtHKT1;1*. No difference was observed between the AOH B03 and AOH B03 *UAS_GAL4_:AtHKT1;1* lines (Figure S4D). Furthermore, calculated unidirectional efflux and transfer of Na^+^ from the leaf sheath to the leaf blade, were not altered in response to the *AtHKT1;1* expression (Figures S4A, S4B and S4C).

Wild-type rice (cv. Nipponbare) was also transformed with a construct containing the CaMV35S promoter fused to the *AtHKT1;1* gene to produce rice plants expressing *AtHKT1;1* constitutively. Many of these plants died in tissue culture, and those that did survive were stunted and generally sterile when grown on soil. Consequently, the plants constitutively overexpressing *AtHKT1;1* were abandoned at this point, as no further analysis was possible.

### Cell-specific overexpression of *AtHKT1;1* increases expression of native *AtHKT1;1* in Arabidopsis and *OsHKT1;5* in rice which leads to decreased shoot Na^+^ accumulation

**Figure 3:**
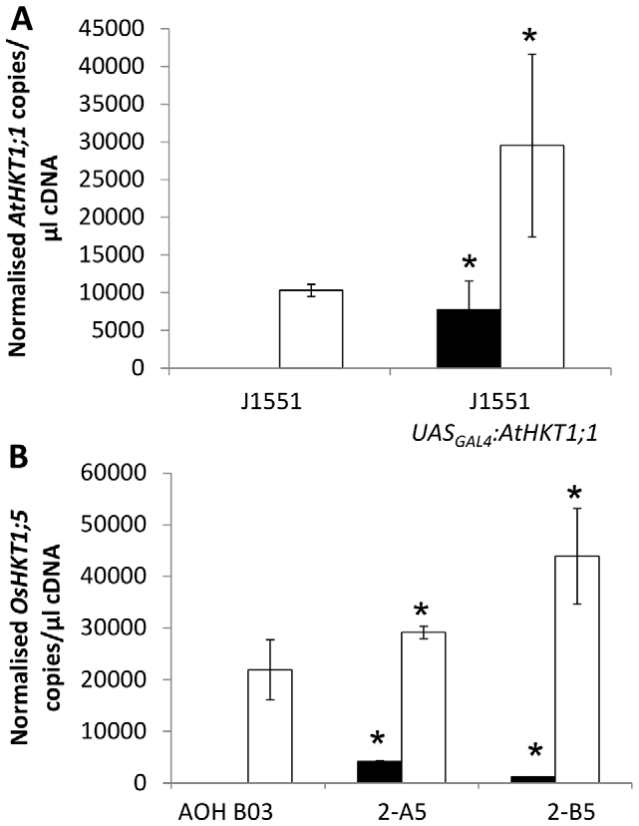
Root expression of *HKT* genes measured using quantitative reverse transcriptase PCR. (A) Normalized measurements of the expression of the *AtHKT1;1* transgene (black bars) and the endogenous *AtHKT1;1* (open bars) in the roots of J1551 and T2 J1551 *UAS_GAL4_:AtHKT1;1* plants (n = 12, error bars represent SEM). Statistical significance from the J1551 line was determined for each gene using Student's t-test, *P<0.05. (B) Normalized measurements of the expression of the *AtHKT1;1* transgene (black bars) and the *OsHKT1;5* gene (open bars) within the roots of AOH B03 and two independently transformed T2 AOH B03 *UAS_GAL4_:AtHKT1;1* lines (2-A5 and 2-B5) (n = 4, error bars represent SEM). Statistical significance from the AOH BO3 line for each gene was determined using Student's t-test, *P<0.05.

Pleiotropic regulation of other genes following misexpression of a gene-of-interest is commonly observed [e.g. 16] and it is possible that a component of the improved salinity tolerance in the transgenic lines may also relate to such an event. Stelar expression of *AtHKT1;1* in Arabidopsis and *OsHKT1;5* in rice regulates the amount of Na^+^ transferred to the shoot [3], [5], [6], [9]. Therefore, expression of the native *HKT* genes in the roots transgenic lines was examined as there was a decrease in root-to-shoot transfer of Na^+^ in these lines. To distinguish transgene copies of *AtHKT1;1* from native *AtHKT1;1* in transgenic Arabidopsis, unique sets of primers were designed (primer sequences are listed in Table S3). The reverse primer for the transgene *AtHKT1;1* was located on a unique restriction site sequence between *AtHKT1;1* and the *nos* terminator sequence, the reverse primer for the native *AtHKT1;1* was located in the 3′ untranslated region of the native *AtHKT1;1* gene. Use of these primer sets revealed a significant increase in transcript abundance of the native *AtHKT1;1* in response to the expression of the transgenic *AtHKT1;1* in the epidermal and cortical root cells (Figure 3A). Analysis of the rice lines for expression of the *AtHKT1;1* transgene indicated a difference in root *AtHKT1;1* expression between the two AOH B03 *UAS_GAL4_:AtHKT1;1* lines (Figure 3B). Similar analysis of the rice AOH B03 *UAS_GAL4_:AtHKT1;1* lines revealed that *OsHKT1;5* was significantly more abundant compared with AOH B03 under conditions of salinity stress (Figure 3B). No other consistent changes in expression between both independent AOH B03 *UAS_GAL4_:AtHKT1;1* lines and the AOH B03 background line of any other member of the *OsHKT* gene family were observed (Figure S5). It should be noted that *OsHKT1;2* is reported to be a pseudogene in *japonica* rice [17] and *OsHKT2;2* is not present in *japonica* rice [17], thus expression of these genes was not examined.

### Outer root cells of transgenic rice overexpressing *AtHKT1;1* accumulate more Na^+^

**Figure 4:**
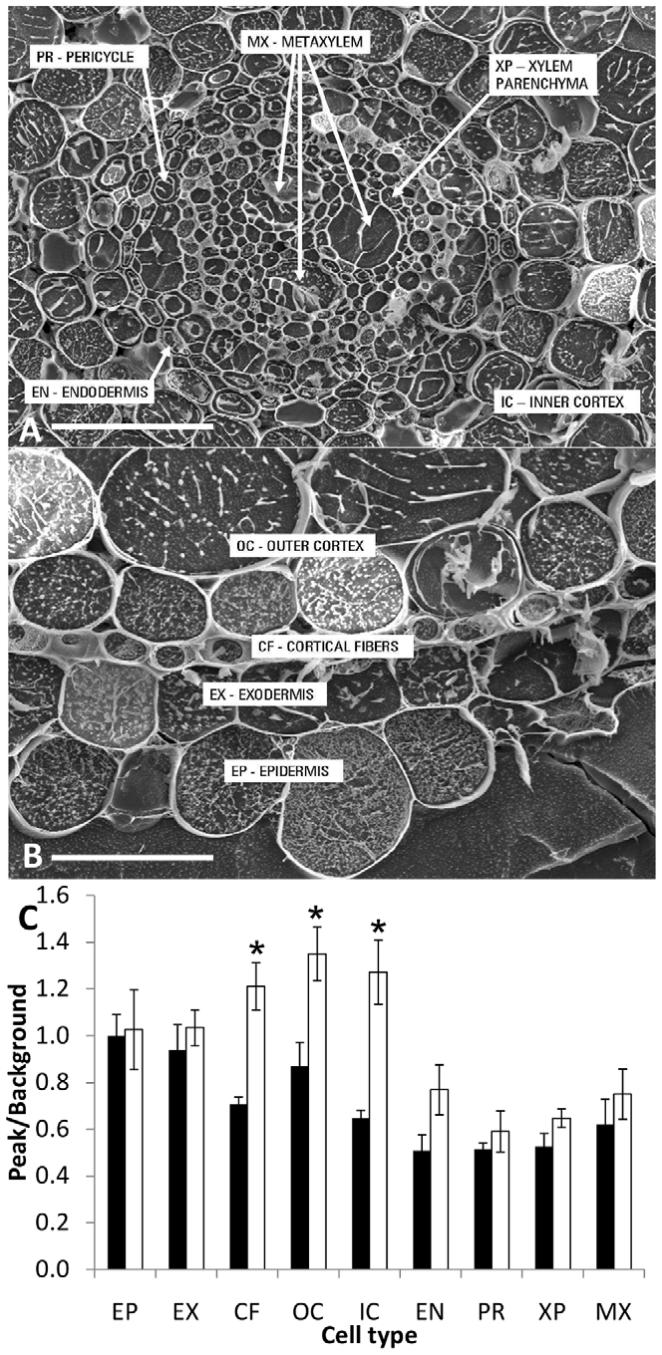
Scanning electron microscopy (SEM) images and measurements of amount of Na^+^ in specific root cell-types. SEM images of the (A) stelar cell-types and (B) outer cell-types of the rice root [Scale bars  = 100 µm in (A) and 20 µm in (B)]. (C) EDAX measurements of the amount of Na^+^ (software measurement of peak/background) present in 9 root cell-types of AOH B03 (black bars) and an AOH B03 *UAS_GAL4_:AtHKT1;1* line (2-B5) (open bars) including: EP - epidermis, EX - exodermis, CF – cortical fibres, OC – outer cortex, IC – inner cortex, EN - endodermis, PR - pericycle, XP – xylem parenchyma, MX - metaxylem. Each bar is an average of measurements made on three cells from each cell-type in the roots of three independent plants grown in a solution containing 50 mM Na^+^ for 5 d (error bars represent SEM). Statistical significance from the AOH BO3 line was determined using Student's t-test, *P<0.05.

Rice root [Na^+^] was higher in the AOH B03 *UAS_GAL4_:AtHKT1;1* line (2-B5) compared to AOH B03, therefore to examine where the extra Na^+^ was stored cryo-scanning electron microscopy and x-ray microanalysis was performed on cell-specific vacuolar contents [9]. Given that extra Na^+^ was likely to be retrieved from the transpiration stream by the native stelar-localized HKT transporters, the aim was to determine where in the root tissue this Na^+^ was being stored. Since the expression of *GFP* (hence, *AtHKT1;1*) was observed in the cortical cells immediately behind the root apex, this tissue was analysed (Figures 4A and 4B). Interestingly, the analysis revealed that the cortical fibres, outer cortex and inner cortex but not the epidermal cells of the AOH B03 *UAS_GAL4_:AtHKT1;1* lines contained significantly higher Na^+^ contents than AOH B03 (Figures 4A–C). It should be noted the AOH B03 *UAS_GAL4_:AtHKT1;1* line (2-B5) chosen for this analysis had the lower whole root Na^+^ concentration of the two independent lines (Figure 2D). The AOH B03 *UAS_GAL4_:AtHKT1;1* lines contained less K^+^ in all cell-types than AOH B03 (Figure S6).

### Increased expression of *AtAVP1* in Arabidopsis, and *OsOVP4, OsOVP5, OsOVP6* in rice leads to increased vacuolar sequestration of Na^+^

**Figure 5:**
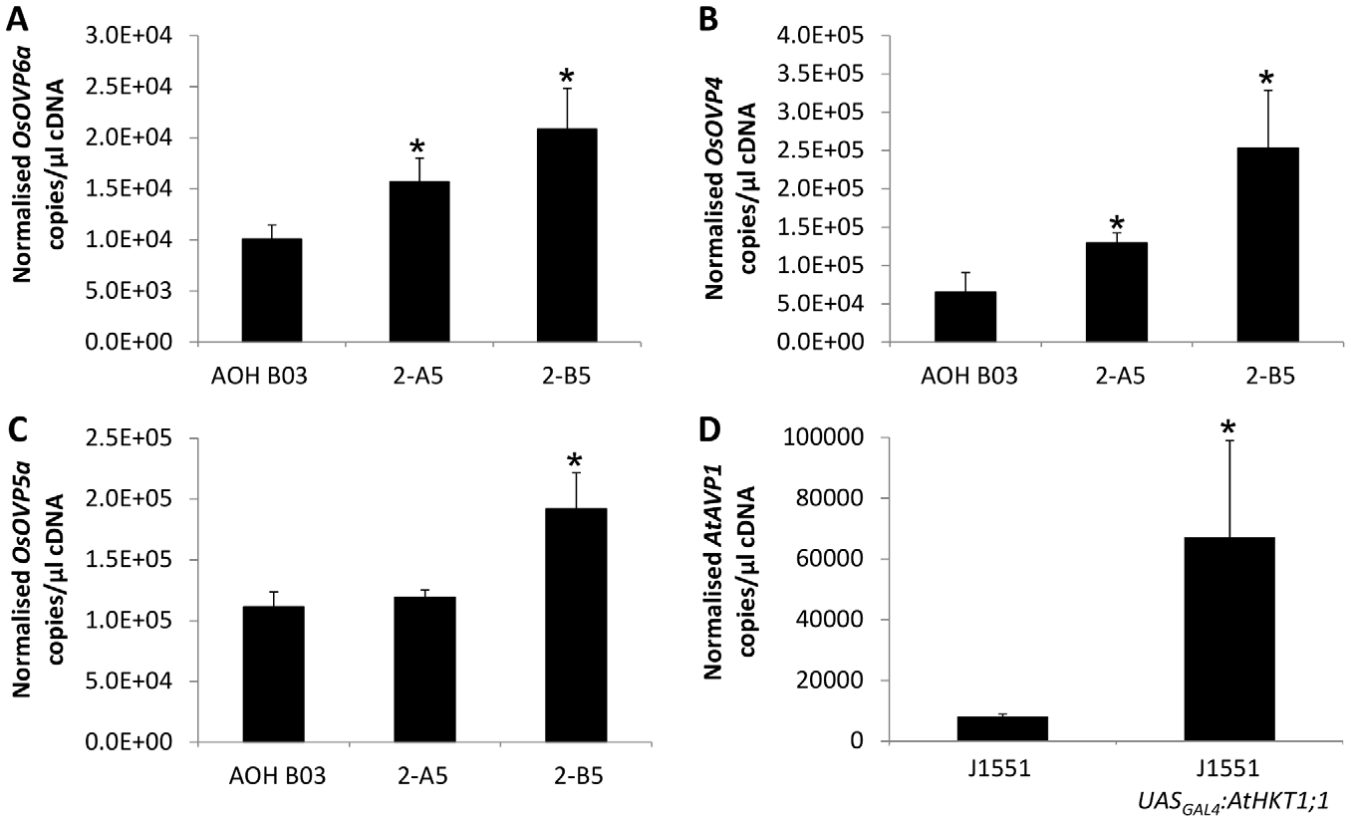
Root expression of vacuolar H^+^-pyrophosphatase genes measured using Quantitative Reverse Transcriptase PCR. Normalized measurements of the expression of (A) *OsOVP4*, (B) *OsOVP5a* and (C) *OsOVP6a* within the roots of AOH B03 and two independently transformed T2 AOH B03 *UAS_GAL4_:AtHKT1;1* lines (2-A5 and 2-B5) (n = 4, error bars represent SEM). Statistical significance from the AOH BO3 line was determined using Student's t-test, *P<0.05. (D) Normalized measurements of the expression of the *AtAVP1* gene in the roots of J1551 and T2 J1551 *UAS_GAL4_:AtHKT1;1* plants (n = 12, error bars represent SEM). Statistical significance from the J1551 line was determined using Student's t-test, *P<0.05.

The expression of *AtHKT1;1* in the cortical cells of the rice roots led to an increase in the vacuolar [Na^+^] of those cells. This may be expected as AtHKT1;1 is the protein that catalyses influx of Na^+^ across the plasma membrane and to avoid damage to cytoplasmic contents, plants must sequester Na^+^ into vacuoles. Two proteins are associated with vacuolar sequestration: the vacuolar Na^+^/H^+^ antiporter, NHX1, which transports Na^+^ into vacuoles; and the vacuolar H^+^-pyrophosphatase, which provides the electrochemical potential necessary to drive the NHX1-mediated movement of Na^+^ into vacuoles by moving protons into the vacuole. *OsNHX1* (Os07g47100) expression showed no upregulation in Na^+^ treated rice roots of AOH B03 *UAS_GAL4_:AtHKT1;1* compared with AOH B03 (Figure S7). Interestingly, expression of *OsOVP4* (Os02g55890), *OsOVP5* (Os02g09150) and *OsOVP6* (Os01g23580) in Na^+^ treated roots from the AOH B03 *UAS_GAL4_:AtHKT1;1* lines was significantly higher than in AOH B03 (Figures 5A, 5B and 5C) while expression of *OsOVP1* (Os06g43660), *OsOVP2* (Os06g08080) and *OsOVP3* (Os05g06480) was decreased or unchanged in response to Na^+^ treatment between the AOH B03 *UAS_GAL4_:AtHKT1;1* and AOH B03 lines (Figure S8).

In Arabidopsis, no difference in root expression of *AtNHX1* (At5g27150) was observed between J1551 and J1551 *UAS_GAL4_:AtHKT1;1* plants, but expression of *AtAVP1* (At1g15690) was significantly higher in the J1551 *UAS_GAL4_:AtHKT1;1* plants (Figure 5D).

## Discussion

The expression of *AtHKT1;1* specifically within the outer cells of the root led to reduced Na^+^ in the shoot in both Arabidopsis and rice. The data from rice experiments revealed that this reduction in shoot Na^+^ resulted from a decreased root-to-shoot flux of Na^+^, and led to increased fresh weight under salinity stress. The decrease in root-to-shoot flux of Na^+^ is related to increased gene expression of the HKT transporter responsible for retrieval of Na^+^ from the transpiration stream and the increased sequestration of Na^+^ in the root cortical cells. This is facilitated by *AtHKT1;1* overexpression in these cells and the pleiotropic increase in transcript abundance of the vacuolar H^+^-translocating pyrophosphatases. However, the sequence of events underlying these observations is less clear.

One possible hypothesis is that the expression of *AtHKT1;1* in outer root cells causes upregulation of *AtHKT1;1* in Arabidopsis and *OsHKT1;5* in rice. The mechanism for this is unknown. *OsHKT1;5* transcript abundance was increased even in control conditions, when root Na^+^ is low, which points to a signalling mechanism that does not require high Na^+^. One possible mechanism could involve the movement from the cortex of small RNAs [18] upregulating *AtHKT1;1/OsHKT1;5* transcription or preventing transcript degradation.

The consequence of increased stelar HKT abundance is an increased retrieval of Na^+^ from the transpiration stream, which must then be removed from the stelar cells to avoid Na^+^ toxicity in these relatively small cells which have only a small vacuole. Thus, the extra Na^+^ is transported back across the endodermis to the cortex of the root, and stored in the extra Na^+^ sink created by the cortical cells expressing *AtHKT1;1*. In order to protect the cytoplasm from Na^+^ toxicity, the accumulated Na^+^ is then further sequestered into the large vacuoles of the cortical cells. Movement into the vacuoles occurs through the Na^+^/H^+^ antiporter, energized by the increase in the electrochemical potential difference for H^+^ resulting from the increased H^+^-pyrophosphatase expression observed (and presumably function). Such an increased capacity for Na^+^ accumulation was described previously [9] in response to direct over-expression of *AtHKT1;1* in the stele and appears to be an important component of limiting Na^+^ transport to the shoot in addition to Na^+^ retrieval from the transpiration stream.

Constitutive overexpression of the *AtHKT1;1* gene was detrimental to plant growth and expression of the gene in stelar tissue did not improve salinity tolerance of rice, thus this study demonstrates the value of cell type-specific expression of Na^+^ transporters for engineering salinity tolerance in important crop plants. A further question arising from our work is why the expression of *AtHKT1;1* in the stelar cells of Arabidopsis improved Na^+^ exclusion and salinity tolerance of Arabidopsis [9], but had no effect or even a detrimental effect on the transgenic rice in this study. Many differences could be cited for this discrepancy including the different stelar cell types expressing *AtHKT1;1* in the previous study [9] and this one. As well, the morphology of Arabidopsis and rice roots are very different, thus creating a different radial root Na^+^ transport scenario for the two species. Finally, there are clear differences in the makeup of the *HKT* family between the two species (and many other important gene families) which could have an important impact on the results of seemingly analogous experiments. This highlights the importance of isolating species specific genetic elements which will enable the driving of cell type-specific expression of transgenes.

In conclusion, expression of *AtHKT1;1* in the outer cells of the root in both Arabidopsis and rice led to lower shoot Na^+^ concentrations and improved salinity tolerance. In rice, this result appears to be due to an upregulation of *OsHKT1;5* expression, as well as an increase in the Na^+^ sequestration capacity of the cortical cells due to increased expression of rice vacuolar H^+^-translocating pyrophosphatases. Several important questions follow from this study and will form the basis of future research. The mechanism leading to the upregulation of the native HKT genes when *AtHKT1;1* is expressed in the outer root layers requires further analysis. Deep sequencing of the small RNA population of these roots may reveal an important regulatory molecule. Further work will also be required to determine whether the transgenic lines produced in this study have increased yield in the field when challenged by salinity stress. Regardless, expressing genes in a cell type-specific manner has improved the salinity tolerance of rice and may be useful in improving tolerance to a wide variety of crop stresses including ion toxicities, mineral deficiencies and potentially even biotic stresses of the roots or leaves.

## Materials and Methods

### Plant Materials and Growth Conditions

*Arabidopsis thaliana* GAL4-GFP enhancer trap line J1551 was produced by Dr. J. Haseloff (Univeristy of Cambridge) in the C24 ecotype background. The rice GAL4-GFP enhancer trap lines AOH B03 and ASG F03 were obtained from a library described previously [12]. Details on growth conditions can be found in Methods S1.

### Microscopy

Fluorescence and confocal microscopy of Arabidopsis and rice was performed as previously described [9], [12], however 30 µm root sections were produced using a Leica VT 1200 S Vibrating Microtome (Leica, Germany). Cryo-scanning electron microscopy and x-ray microanalysis was performed as described previously [9] with modifications described in Methods S1.

### DNA Constructs and Transformation

Binary vectors used to transform Arabidopsis have been described previously [8]. Details on binary vectors used to transform rice can be found in SI Materials and Methods. Rice transformation is as described previously [13], [14] with minor modifications noted in Methods S1.

### GUS Staining

Staining of tissues for visualization of *uidA* expression was performed as described previously [9], [12].

### Quantitative RT-PCR (Q-PCR)

Arabidopsis tissue samples were collected from the transformed plants when the plants were 5 weeks old. Approximately 15 mg of root and shoot tissue was collected from plants previously selected by spraying with phosphinotricin. Samples were freeze-dried and RNA was extracted from tissue powder using the Pure-Link 96 RNA Kit (Invitrogen) following the manufacturer's instructions. The RNA was treated using the DNA-free kit (Ambion) and cDNA was synthesized using SuperScript III First-Strand Synthesis System (Invitrogen) according the manufacturer's instructions.

Rice root tissue was ground under liquid nitrogen and total RNA was extracted from approximately 100 mg of ground tissue using the TRIzol reagent (Invitrogen) according to the manufacturer's instructions.

Q-PCR was performed as described previously [19] with modifications noted in Methods S1 and using primers in Table S3.

### Tissue Elemental Analysis

Arabidopsis tissue was analyzed for elemental content via ICP-AES as described previously [9]. Rice leaf blades, sheaths and roots from individual plants were harvested into 50 mL Falcon tubes for Na^+^ and K^+^ accumulation analysis. Fresh weights of samples were taken, the tissue was dried in an oven at 70°C for 12 h and dry weights were recorded. Samples were digested in 10 mL of 1% nitric acid for 4 h at 80°C. Digested samples were then analysed using a flame photometer (Sherwood Scientific Ltd., Cambridge, U.K.) for Na^+^ and K^+^ content. Values from the flame photometer were converted into Na^+^ and K^+^ concentration values on a tissue water basis (millimolar).

### ^22^Na^+^ Fluxes

Experiments were conducted on rice plants that had been pretreated with 30 mM NaCl in the hydroponic solution prior to analysis in an influx solution containing 30 mM NaCl and labelled with 0.05 µCi/ml ^22^Na^+^. Experiments were conducted essentially as previously described [20] with modifications described in Methods S1.

## Supporting Information

Methods S1 Additional Materials and Methods.
(0.06 MB DOC)

Table S1 ICP-MS measurements of the concentration (in mg/kg) of several common elements in the leaf tissue of J1551 compared with independent T1 J1551 *UAS_GAL4_:AtHKT1;1* lines. Plants were grown on 2 mM NaCl.
(0.03 MB DOC)

Table S2 ICP-AES measurements of the concentration (in mg/kg) of several common elements in the leaf tissue of J1551 (‘Background’) compared with independent T2 J1551 *UAS_GAL4_:AtHKT1;1* families. Plants were grown on 5 mM NaCl.
(0.04 MB DOC)

Table S3 List of primers used for vector construction and Q-PCR analysis in Arabidopsis and rice. Forward and reverse primers are listed and the gene identifier for each gene analysed by Q-PCR is provided.
(0.05 MB DOC)

Figure S1 Images of the rice GAL4-GFP enhancer trap line ASG FO3. (A + B) Confocal laser microscope images of line ASG F03 showing GFP fluorescence specifically within the root xylem parenchyma [Scale bars  = 100 µm in (A) and 100 µm in (B)]. Tissue was stained with propidium iodide. GFP and propidium iodide images were captured separately and overlaid to create the composite image. Tissues labelled include the epidermis (ep), cortex (c), endodermis (en), xylem parenchyma (xp) and metaxylem (mx).
(2.65 MB TIF)

Figure S2 Fresh weight (FW) measurements and tissue Na^+^ and K^+^ concentration of T1 rice lines. Lines express *AtHKT1;1* within the root cortical cells (AOH B03 *UAS_GAL4_:AtHKT1;1*) or the root xylem parenchyma cells (ASG F03 *UAS_GAL4_:AtHKT1;1*) and are compared with their respective background lines (AOH B03 and ASG F03). Measurements of FW of (A) shoots and (B) roots of control (black bars) and salinity stressed (open bars) rice plants grown for 5 d on 80 mM NaCl (n = 8, error bars represent SEM). Statistical significance between treatments for each line was determined via the Student's t-test, *P<0.05. Tissue concentration of (C) Na^+^ and (D) K^+^ in roots (black bars) and shoots (open bars) of rice plants grown for 5 d on 80 mM NaCl (n = 8, error bars represent SEM). Statistical significance from the respective control line for each tissue was determined via the Student's t-test, *P<0.05.
(0.21 MB TIF)

Figure S3 Fresh weight measurements of T2 rice plants. The FW was measured in (A) shoots and (B) roots of rice plants grown in control conditions (black bars) or in salinity stress conditions (open bars). (C) Concentration of K^+^ in the roots (black bars) and shoots (open bars) under salinity stress conditions. AOH B03 and two independent T2 AOH B03 *UAS_GAL4_:AtHKT1;1* lines (2-A5 and 2-B5) were grown for 5 d on nutrient solution containing 80 mM NaCl (n = 8, error bars represent SEM). Statistical significance from the AOH BO3 line was determined via the Student's t-test, *P<0.05.
(0.12 MB TIF)

Figure S4 Flux measurements of Na^+^ inferred using the radiotracer ^22^Na^+^. Measurements were taken in AOH B03 and two independent T2 AOH B03 *UAS_GAL4_:AtHKT1;1* lines (2-A5 and 2-B5) grown for 3 d prior to experiment on nutrient solution containing 30 mM Na^+^ (n = 8, error bars represent SEM). (A) Measurement of the unidirectional influx of ^22^Na^+^ into non-excised rice roots exposed to an influx solution containing 30 mM Na^+^ for 2 min. (B) Root efflux of ^22^Na^+^ calculated by subtracting the net influx of ^22^Na^+^ into the plant (after 1 h exposure of the roots to a solution containing 30 mM Na^+^) from the unidirectional influx of ^22^Na^+^ (after a 2 min exposure of the roots to a solution containing 30 mM Na^+^). (C) Measurement of the sheath-to-blade transfer of ^22^Na^+^ expressed as a percentage of the total amount of ^22^Na^+^ in the shoot tissue that is contained within the leaf blades after exposing the roots of intact plants to a solution containing 30 mM Na^+^ for 1 h. (D) Measurement of the unidirectional influx of ^22^Na^+^ into rice roots excised immediately prior to experiment and exposed to an influx solution containing 30 mM Na^+^ for 2 min.
(0.19 MB TIF)

Figure S5 Expression of *OsHKT* family members measured using quantitative reverse transcriptase PCR. Included are AOH B03 and AOH B03 *UAS_GAL4_:AtHKT1;1* lines 2-A5 and 2-B5. Normalized expression of (A) *OsHKT1;1*, (B) *OsHKT1;3*, (C) *OsHKT1;4*, (D) *OsHKT2;1*, (E) *OsHKT2;3* and (F) *OsHKT2;4* in the roots of rice plants grown in 80 mM NaCl for 5 d. Each bar represents an average of four replicates (error bars represent SEM). Statistical significance from the AOH BO3 line was determined using Student's t-test, *P<0.05.
(0.43 MB TIF)

Figure S6 EDAX measurements of the amount of K^+^ (software measurement of peak/background) present in 9 root cell-types of rice. Lines include AOH B03 (black bars) and a AOH B03 *UAS_GAL4_:AtHKT1;1* line (2-B5) (open bars) and cell types are: EP - epidermis, EX - exodermis, CF - cortical fibres, OC - outer cortex, IC - inner cortex, EN - endodermis, PR - pericycle, XP - xylem parenchyma, MX - metaxylem. Each bar is an average of measurements made on three cells from each cell-type in the roots of three independent plants grown in a solution containing 50 mM Na^+^ for 5 d (error bars represent SEM). Statistical significance from the AOH BO3 line was determined via the Student's t-test, *P<0.05.
(0.10 MB TIF)

Figure S7 Expression of the Na^+^/H^+^ antiporter gene *OsNHX1* measured using quantitative reverse transcriptase PCR. Included are AOH B03 and AOH B03 *UAS_GAL4_:AtHKT1;1* lines. Normalized expression of the vacuolar H^+^-pyrophosphatases (A) *OsOVP1*, (B) *OsOVP2* and (C) *OsOVP4* in the roots of rice plants grown in 80 mM NaCl for 5 d. Each bar represents an average of four replicates (error bars represent SEM). Statistical significance from the AOH BO3 line was determined using Student's t-test, *P<0.05.
(0.05 MB TIF)

Figure S8 Expression of several rice vacuolar H^+^-pyrophosphatase genes measured via quantitative reverse transcriptase PCR. Included are AOH B03 and AOH B03 *UAS_GAL4_:AtHKT1;1* lines. Normalized expression of the vacuolar H^+^-pyrophosphatases (A) *OsOVP1*, (B) *OsOVP2* and (C) *OsOVP4* in the roots of rice plants grown in 80 mM solution for 5 d. Each bar represents an average of 4 replicates (error bars represent SEM). Statistical significance from the AOH BO3 line was determined via the Student's t-test, *P<0.05.
(0.19 MB TIF)
