# Supplementary material for: Improved Salinity Tolerance of Rice Through Cell Type-Specific Expression of AtHKT1;1
Source: PLoS One. 2010 Sep 3;5(9):e12571. doi: 10.1371/journal.pone.0012571 (PMC2933239; doi:10.1371/journal.pone.0012571)
Supplement: Methods S1 — Additional Materials and Methods. (0.06 MB DOC) [file pone.0012571.s001.doc]

**Methods S1**

***GAL4-GFP seed source***

An enhancer trap line with GFP fluorescence in root tissue was selected from an *Arabidopsis* *thaliana* library of GAL4-GFP expressing plants. A catalogue of this enhancer trap library is published at [http://www.plantsci.cam.ac.uk/Haseloff/IndexGAL4.htm1](http://www.plantsci.cam.ac.uk/Haseloff/IndexGAL4.htm1)). These lines were generated by Dr. J. Haseloff (University of Cambridge), who kindly provided the seed for this research, including the wild-type ecotype C24. Experiments were performed using the J1551 line that is in the ecotype C24 and shows GFP fluorescence specifically in the root epidermis and cortex. Line J1551 was determined to have a single insertion of the enhancer trap in the T3 generation using Southern blotting as previously described [1]. One minor difference was that the genomic DNA was digested using *BglII* and *SpeI* (New England Biolabs).

Two GAL4-GFP enhancer trap lines were used in the rice transformation. The first line, AOH B03, has root cortex specific GFP fluorescence. The second line, ASG F03, has root xylem parenchyma specific GFP fluorescence. Detailed descriptions of the GAL4-GFP enhancer trap patterns can be found in an online database (http://129.127.183.5/fmi/iwp/res/iwp_home.html) and their development has been described previously [2].

***Plant growth***

Arabidopsis plants were grown in sterile culture plates or artificial soil mix as previously described [1]. Salt was applied in the nutrient watering solution twice per week, at a concentration of 2 mM (for the T1 plants) and 5 mM (for the T2 plants), so the concentration of NaCl built up as the plants grew.

All rice experiments used the following protocol for seed preparation and seedling growth. Prior to imbibition, seeds were sterilized with 70% ethanol for one minute, and excess ethanol was decanted. Sodium hypochlorite (30% of the purchased product – e.g. White King – which generates about 1% available chlorine) plus a few drops of Tween20 was added to the seeds for 30 minutes, then the seeds were washed thoroughly (at least five times) with RO water. Surface sterilized seeds were placed in deep cell culture dishes (Petri dishes) on filter paper with 5 mL RO water. The dishes were wrapped with Parafilm and placed in a growth chamber with 28°C days and 24°C nights (16 h full light and high humidity). After five days of growth on dishes, seedlings were transplanted to 10 L hydroponics boxes and were supported by cut off 1.5 mL Eppendorf tubes. To minimize nutrient depletion and microbial growth, the nutrient solution was replaced with fresh nutrient solution after the first week of growth, and then replaced every three to four days, depending on the size and number of plants per tank. The seedlings were allowed 7 d to grow on nutrient solution before stress application. The nutrient solution comprised 5 mM NH_4_NO_3_; 5 mM KNO_3_; 2 mM Ca(NO_3_)_2_*4H_2_O; 2 mM MgSO_4_*7H_2_O; 0.1 mM KH_2_PO_4_; 0.05 mM NaFe(III)EDTA; 50 µM H_3_BO_3_; 5 µM MnCl_2_*4H_2_O; 5 µM ZnSO_4_*7H_2_O; 0.5 µM CuSO_4_*5H_2_O; and 0.1 µM Na_2_MoO_3_; RO water; pH 5.5. The seedlings were grown in a growth chamber with tightly controlled growth conditions of 28°C days, 26°C nights, 16 h of full light (high-pressure sodium lamps), and 90% humidity during the days and 80% humidity at night. Plants were grown on nutrient solution without added Na^+^ for 8 d, then on 10 mM NaCl for 4 d, then on 20 mM for 2 d, then on 40 mM for 2 d and 80 mM for 5 d. Plants were separated into leaf blades, leaf sheaths, roots and a subsample of root tissue was weighed and immediately frozen in liquid nitrogen for future RNA extraction and Q-PCR analysis.

***Microscopy***

Fluorescence microscopy and confocal scanning laser microscopy of Arabidopsis were performed as previously described [1].

Rice root GFP fluorescence was detected using a Leica MZ FLIII fluorescent stereomicroscope with a GFP2 filter set (excitation 480/40 nm, barrier filter LP510 nm) and images captured using a DC300F digital camera and IM50 software, version 1.20 (Leica Microscopie Systems, Heerbrugg, Switzerland). For confocal laser scanning microscopy, roots were immersed in propidium iodide (10 μg ml^-1^) for 5 min to outline cell walls and provide a counter fluorophore to the GFP. A Zeiss Axioskop 2 mot plus LSM5 PASCAL laser scanning microscope, equipped with an argon laser, and was used for imaging (Carl Zeiss, Jena, Germany). GFP was detected using an excitation of 488 nm and an emission of 505-530 nm, propidium iodide was detected using an excitation of 543 nm and a LP 560 nm emission filter.  Images were captured using PASCAL version 3.2 SP2 software (Carl Zeiss) and then overlaid to create composite images. Cross-sections were obtained by fixing roots to an apple with superglue and 30 μm sections were cut using a Leica VT 1200 S Vibrating Microtome (Leica, Germany). Sections were floated on water and moved to stain in propidium iodide (10 μg ml^-1^) for 5 min and rinsed twice in deionised water. Sections were then placed in water on a slide and imaged as previously above.

***DNA constructs***

The construction of the binary vectors containing UAS_GAL4_:*uidA* and UAS_GAL4_:*AtHKT1;1* for transformation of Arabidopsis is described previously [1].

Constructs used to transform rice were developed as follows. The pGreen-UAS_GAL4_-nos plasmid [1] was used as a template in PCR with the oligonucleotides UAS_GAL4_*KpnI*F and UAS_GAL4_*AscI*R (primer sequences are presented in Table S3) to amplify the UAS_GAL4_ region. Primer UAS_GAL4_*KpnI*F included a *KpnI* restriction enzyme site at the 5’ terminus and the primer UAS_GAL4_*AscI*R included an *AscI* restriction enzyme site for subsequent cloning steps. The purified PCR product was digested with *KpnI* and *AscI*. The pMDC100 plasmid (Ueli Grossniklaus - University of Zurich) was digested with *KpnI* and *AscI* the compatible UAS_GAL4_ fragment was ligated into the vector. pMDC32 (Ueli Grossniklaus - University of Zurich) was used as a template in PCR using NosTF*AleI* and NosTR*PacI* (primer sequences are presented in Table S3) to amplify the nopaline synthase (Nos) terminator. The PCR product and the pMDC100 plasmid (containing the UAS_GAL4_ fragment) were digested using *AleI* and *PacI* restriction enzymes. The nos terminator PCR fragment described above was then ligated into the pMDC100+UAS_GAL4_ vector to generate pMDC100+UAS_GAL4_+nos. The *AtHKT1;1* gene (At4g10310) was PCR amplified from pGreen-UAS_GAL4_-*AtHKT*-nos [1] using primers HKT1Fwd and HKT1R (primer sequences are presented in Table S3) and was cloned into the vector pCR8/GW/TOPO TA (Invitrogen) according to the manufacturer’s instructions. The *AtHKT1;1* gene was recombined into the UAS_GAL4_ destination vector and the *AtHKT1;1* gene was recombined into the pMDC32 destination vector using LR Clonase II (Invitrogen) according to the manufacturer’s instructions.

***Transformation***

Arabidopsis J1151 line plants were transformed using the floral dip method [3]. Transformation of the binary vectors into *Agrobacterium* was performed as described previously [1]. Transformed plants were resistant to phosphotricin.

The binary vectors for rice transformation were transformed into the AGL1 or AGL0 strains of *Agrobacterium* using the ‘freeze-thaw’ transformation protocol [4]. *Agrobacterium*-mediated transformation of scutellum-derived callus was used to produce transgenic rice lines [5,6]. Transformed callus was selected on media containing hygromycin (50 mg l^-1^) in the case of the CaMV35S binary vector or geneticin (200 mg l^-1^) in the case of the UAS_GAL4_ binary vector. Regenerated plantlets were removed from jars and the shoots and roots were trimmed (leaving 2 cm of shoot and 1 cm of root). Regenerants were placed into Jiffy peat pots in trays in water and covered with a plastic dome. Trays were kept in a glasshouse with average temperatures of 30°C days and 20°C nights, full sun, and an average humidity of 60%. After 15 d regenerants were planted into soil (UC Davis mix) in pots and grown to maturity in the glasshouse.

***Quantitative RT-PCR (Q-PCR)***

Arabidopsis tissue samples were collected from the transformed plants when the plants were 5 weeks old. Approximately 15 mg of root and shoot tissue was collected from plants previously selected using spraying with phosphinotricin. Samples were freeze-dried and RNA was extracted from tissue powder using the Pure-Link 96 RNA Kit (Invitrogen) following the manufacturer’s instructions. The RNA was treated using the DNA-free kit (Ambion) and cDNA was synthesized using SuperScript III First-Strand Synthesis System (Invitrogen) according the manufacturer’s instructions.

Rice root tissue was ground under liquid nitrogen and total RNA was extracted from approximately 100 mg of ground tissue using the TRIzol reagent (Invitrogen) according to the manufacturer’s instructions.

The protocol for Q-PCR analysis was the same for Arabidopsis and rice. The RNA was treated with rDNase I using a DNA-*free* kit (Ambion) and RNA integrity was checked on a 1.2% agarose gel (w/v). cDNA synthesis was performed on 1 µg of RNA with a 19 mer polyT primer and a SuperScript III reverse transcriptase kit (Invitrogen) according to the manufacturer’s instructions. Q-PCR was carried out essentially as outlined previously [7] with the following modifications. Preparation of Q-PCR standard: between four and six 20 µL PCRs were combined for purification by HPLC [8] using a HELIX DNA DVB 50 x 3.0 mm monolithic polymer reversed phase column (Varian Inc.). Chromatography was performed as follows. The buffers were buffer A: 100 mM triethylammonium acetate (Applied Biosystems), 0.1 mM EDTA and buffer B: 100 mM triethylammonium acetate, 0.1 mM EDTA, 75% acetonitrile. The gradient was as follows: time 0 minute, 10% buffer B; time 6 minutes 21.5% buffer B; time 7 minutes, 21.5% buffer B; time 8 minutes, 10% buffer B; time 12 minutes, 10% buffer B. The flow rate was 0.45 mL/min and the temperature was 50°C throughout the chromatography. Q-PCR protocol: three replicates of each of the seven standard concentrations were included with every Q-PCR experiment together with a minimum of three no-template controls. Q-PCR experiments were assembled by the liquid handling robot CAS-1200 robot (Corbett Robotics). Three replicate PCRs for each of the cDNAs were included in every run containing as follows: 2 µL cDNA solution, the diluted standard or water was used in a reaction containing 5 µL IQ SYBR Green PCR reagent (Bio-rad Laboratories), 1.2 µL each of the forward and reverse primers at 4 µM, 0.3 µL 10x SYBR Green in water and 0.3 µL water. The total volume of this PCR was 10 µL. Reactions were performed in a RG6000 Rotor-Gene Real Time Thermal Cycler (Corbett Research); 3 minutes at 95°C followed by 45 cycles of 1 s at 95°C, 1 s at 55°C, 30 s at 72°C and 15 s at the optimal acquisition temperature for each specific gene product. Five control genes in rice (GaPDh, Actin, Tubulin, PpIase and ElF1) and two control genes in Arabidopsis (GaPDh and Actin) were assessed and the best three rice genes (i.e. with the most stable expression across treatments and genotypes) were chosen for the calculation of the normalisation factor (Actin, Tubulin and ElF1). Q-PCR normalisation was carried out as detailed previously [7,9]. Primer sequences for all control genes and all genes-of-interest are listed in Table S3.

***Tissue elemental analysis***

Arabidopsis shoot tissue was harvested and the shoot ion accumulation was analysed using a CIROS Radial Inductively Coupled Plasma Optical Emission Spectrometer (ICP-AES, Spectro) as described previously [1]. T2 seedlings were genotyped and only plants containing the UAS_GAL4_:*AtHKT1;1* T-DNA were included in the ion accumulation data. Plants were genotyped by PCR amplifying a band using the HKTQFwd and HKTQRev primers listed in Table S3.

Rice leaf blades, sheaths and roots from individual plants were harvested into 50 mL Falcon tubes for Na^+^ and K^+^ accumulation analysis. Fresh weights of samples were taken, the tissue was dried in an oven at 70°C for 12 h and dry weights were recorded. Samples were digested in 10 mL of 1% nitric acid for 4 h at 80°C. Digested samples were then analysed using a flame photometer (Sherwood Scientific Ltd., Cambridge, U.K.) for Na^+^ and K^+^ content. Values from the flame photometer were converted into Na^+^ and K^+^ concentration values on a tissue water basis (millimolar).

***^22^Na^+^ fluxes***

Experiments were conducted using intact plants that had been treated in the hydroponic tanks for 3 d with 30 mM NaCl and 0.5 mM CaCl_2_ (adjusted to compensate for the drop in Ca^2+^ activity from the addition of various Na^+^ concentrations using the program Visual MINTEQ ver 2.40b – KTH, Department of Land and Water Resources Engineering, Stockholm, Sweden) to allow plants to adjust to the treatment Na^+^ concentration. Hydroponic tanks were moved from the growth chamber to the flux bay 1 h prior to the commencement of influx treatments. All experiments were conducted under a fluorescent light bank at 22°C to 26°C. The roots of intact plants were blotted dry after removal from the hydroponic tank. For unidirectional influx measurements in excised roots, the roots were excised from the plant immediately prior to treatment in influx solution. Unidirectional influx measurements were determined on plants placed in the influx solution for 2 min, while root-to-shoot fluxes were determined on plants placed in the influx solution for 1 h. Efflux from roots was calculated by subtracting total influx from unidirectional influx values. Roots were placed in 50 mL of influx solution (30 mM NaCl, 0.5 mM KCl and 0.1 mM CaCl_2_ activity) labelled with 0.05 µCi/ml of ^22^Na^+^ (Amersham/GE Healthcare, Sydney, Australia) by fixing the Eppendorf tube holding the plant to the side of the influx solution vessel using Power Tack adhesive (Acco, Australia). Experiments were conducted on gently rotating shakers (35 rpm) (Ratek, Boronia, Australia) to keep solutions aerated and reduce boundary layer effects. Following influx treatment, intact plants were removed from the influx solution and extra influx solution was allowed to drip off roots. Roots were rinsed quickly in room temperature rinse solution (30 mM NaCl, 0.5 mM KCl and 10 mM CaCl_2_) to displace apoplastically bound ^22^Na^+^ then excised below the seed and rested in tea strainers in two successive rinses in 500 ml of ice cold (4°C) rinse solution (2 min + 2 min) on shakers (35 rpm). Rinse solutions were changed every 4 h. Roots were blotted dry with paper towels and immediately weighed in 6 ml scintillation vials (PerkinElmer, Melbourne, Australia). Four ml of scintillation fluid (Ecolume, MP Biomedicals, Sydney, Australia) was added to each vial and each vial was shaken and measured using a liquid scintillation counter (Beckman Coulter LS6500, Gladesville, Australia). Immediately prior to and immediately following each experiment three 20 µL samples of the influx solution were placed into scintillation vials, scintillation fluid was added and shaken, to enable calculation of specific activity for each influx solution. This allowed conversion of the counts of radioactivity in the roots to an amount of chemical Na^+^, and thus a conversion to a flux. Blank vials were also measured to allow subtraction of background radiation from the measurements of the samples.

***Cryo-scanning electron microscopy and x-ray microanalysis***

The plants were grown in 80 mM Na^+^ for 5 d and were analysed for cell type-specific ionic contents using cryo-scanning electron microscopy and x-ray microanalysis. Roots from three 3 week-old hydroponically-grown rice plants of each line were examined. Roots were cut 5 mm from the root tip and inserted into a hole in a brass stub which supported the root sections to stand vertically for examination in the microscope. Four roots of each genotype (AOH B03 and AOH B03 *UAS_GAL4_:AtHKT1;1*) were placed in the same stub in each experiment with a drop of growth solution surrounding them and snap frozen in liquid N_2_ slush. The frozen specimen was transferred under vacuum to a cryo-microtome where it was cryoplaned using a liquid N_2_ cooled microtome blade. Thus, the actual surface examined was 4 mm from the root tip to ensure that a section without aerenchyma, but expressing the transgene, was examined. The samples were transferred to the specimen stage of the scanning electron microscope and were etched for 1.5 min at -92°C to sublimate some cellular water in order to more easily observe cellular structure. Samples were cooled to -120°C and sputter coated with platinum for 1.5 min to ensure proper electrical conductivity between the specimen and the stub. The coated specimens were then loaded onto the microscope stage (held at a temperature below -150°C) and analysed in a Philips XL 30 Scanning Electron Microscope (Philips Electron Optics) fitted with a CT1500 HF cryotransfer stage (Oxford Instruments) and an EDAX energy-dispersive x-ray detector (EDAX International). XRMA spectra were recorded at a voltage of 10 kV, a working distance of 10 µm and a data collection time of 100 live s. Spectra were analysed with eDXi software (EDAX) and results presented were measurements of peak over background. Data were compiled using XRMAplot (courtesy of Elena Kalashyan and Ute Baumann, ACPFG). Cell-types analysed were epidermis (EP), exodermis (EX), cortical fibres (CF), outer cortex (OC), inner cortex (IC), endodermis (EN), pericycle (PR), xylem parenchyma (XP) and metaxylem (MX). The values are given as the semi-quantitative peak over background values (P/B).

**References**

1. Møller IS, Gilliham M, Jha D, Mayo G, Roy S et al. (2009) Shoot Na^+^ exclusion and increased salinity tolerance engineered by cell type-specific alteration of Na^+^ transport in Arabidopsis. Plant Cell 21: 2163-2178.

2. Johnson AAT, Hibberd JM, Gal CI, Essah PA, Haseloff J et al. (2005) Spatial control of transgene expression in rice (*Oryza sativa* L.) using the GAL4 enhancer trapping system. Plant J 41: 779-789.

3. Weigel D, Glazebrook J (2006) *In* *planta* transformation of Arabidopsis. Cold Spring Harbor Protocols 2006: pdb.prot4668-.

4. Weigel D, Glazebrook J (2006) Transformation of *Agrobacterium* using the freeze-thaw method. Cold Spring Harbor Protocols 2006: pdb.prot4666-.

5. Sallaud C, Meynard D, Boxtel, Jv, Gay C, Bés M et al. (2003) Highly efficient production and characterization of T-DNA plants for rice (*Oryza sativa* L.) functional genomics. TAG 106: 1396-1408.

6. Sallaud C, Gay CI, Larmande P, Bés M, Piffanelli P et al. (2004) High throughput T-DNA insertion mutagenesis in rice: a first step towards *in* *silico* reverse genetics. Plant J 39: 450-464.

7. Burton RA, Shirley NJ, King BJ, Harvey AJ, Fincher GB (2004) The *CesA* gene family of barley. Quantitative analysis of transcripts reveals two groups of co-expressed genes. Plant Phys 134: 224-236.

8. Wong LY, Belonogoff V, Boyd VL, Hunkapiller NM, Casey PM et al. (2000) General method for HPLC purification and sequencing of selected dsDNA gene fragments from complex PCRs generated during gene expression profiling. BioTechniques 28: 776-783.

9. Vandesompele J, De Preter K, Pattyn F, Poppe B, Van Roy N et al. (2002) Accurate normalization of real-time quantitative RT-PCR data by geometric averaging of multiple internal control genes. Genome Biology 3: research0034.0031 - research0034.0011.
