# Supplementary material for: Improved Salinity Tolerance of Rice Through Cell Type-Specific Expression of AtHKT1;1
Source: PLoS One. 2010 Sep 3;5(9):e12571. doi: 10.1371/journal.pone.0012571 (PMC2933239; doi:10.1371/journal.pone.0012571)
Supplement: Table S1 — ICP-MS measurements of the concentration (in mg/kg) of several common elements in the leaf tissue of J1551 compared with independent T1 J1551 UASGAL4:AtHKT1;1 lines. Plants were grown on 2 mM NaCl. (0.03 MB DOC) [file pone.0012571.s002.doc]

**Table S1:** ICP-MS measurements of the concentration (in mg/kg) of several common elements in the leaf tissue of Arabidopsis line J1551 compared with independent T1 J1551 *UAS_GAL4_:AtHKT1;1* lines. Plants were grown on 2 mM NaCl.

| T1 |  | n | Mn | B | Zn | Ca | Mg | Na | K | P |
| --- | --- | --- | --- | --- | --- | --- | --- | --- | --- | --- |
| J1551 | Mean | 12 | 5.0 | 3.9 | 5.0 | 1238 | 408 | 730 | 2599 | 501 |
|  | SEM |  | 0.6 | 1.1 | 0.5 | 68 | 27 | 28 | 132 | 53 |
| J1551 | Mean | 45 | 4.6 | 2.9 | 7.4 | 1282 | 430 | 585 | 2935 | 700 |
| *UAS_GAL4_:AtHKT1;1* | SEM |  | 0.3 | 0.3 | 0.3 | 66 | 10 | 26 | 86 | 20 |
| t-test | *P* value |  | 0.605 | 0.360 | 0.001 | 0.646 | 0.450 | 0.001 | 0.045 | 0.003 |
