# Supplementary material for: Improved Salinity Tolerance of Rice Through Cell Type-Specific Expression of AtHKT1;1
Source: PLoS One. 2010 Sep 3;5(9):e12571. doi: 10.1371/journal.pone.0012571 (PMC2933239; doi:10.1371/journal.pone.0012571)
Supplement: Table S2 — ICP-AES measurements of the concentration (in mg/kg) of several common elements in the leaf tissue of J1551 (‘Background’) compared with independent T2 J1551 UASGAL4:AtHKT1;1 families. Plants were grown on 5 mM NaCl. (0.04 MB DOC) [file pone.0012571.s003.doc]

**Table S2:** ICP-AES measurements of the concentration (in mg/kg) of several common elements in the leaf tissue of Arabidopsis line J1551 (‘Background’) compared with independent T2 J1551 *UAS_GAL4_:AtHKT1;1* families. Plants were grown on 5 mM NaCl.

| T2 Family | Element | Average | Mean ± S.E.M | | *P* value |
| --- | --- | --- | --- | --- | --- |
|  |  | % change | J1551 (n=3) | J1551 *UAS_GAL4_:AtHKT1;1* (n=20) |  |
| HKT-A16 | Na | -37 | 1987±107 | 1260±34 | 0.049 |
|  | K | 19 | 3033±120 | 3615±71 | 0.023 |
| HKT-A22 | Na | -16 | 1523±71 | 1284±18 | 0.042 |
|  | K | 38 | 2800±57 | 3860±49 | 0.026 |
| HKT-A26 | Na | -37 | 2567±38 | 1616±24 | 0.001 |
|  | K | 30 | 3400±68 | 4425±212 | 0.028 |
| HKT-A34 | Na | -19 | 1603±43 | 1294±16 | 0.023 |
|  | K | 45 | 2633±21 | 3819±32 | 0.041 |
