# Supplementary material for: Improved Salinity Tolerance of Rice Through Cell Type-Specific Expression of AtHKT1;1
Source: PLoS One. 2010 Sep 3;5(9):e12571. doi: 10.1371/journal.pone.0012571 (PMC2933239; doi:10.1371/journal.pone.0012571)
Supplement: Table S3 — List of primers used for vector construction and Q-PCR analysis in Arabidopsis and rice. Forward and reverse primers are listed and the gene identifier for each gene analysed by Q-PCR is provided. (0.05 MB DOC) [file pone.0012571.s004.doc]

**Table S3:** List of primers used for vector construction and Q-PCR analysis in Arabidopsis and rice. Forward and reverse primers are listed and the gene identifier for each gene analysed by Q-PCR is provided.

| **Primer Name** | **Gene Identifier** | **Sequence (5’ to 3’)** |
| --- | --- | --- |
| UAS_GAL4_*KpnI*Fwd  UAS_GAL4_*AscI*Rev | N/A | ACGGTACCGCATGCCTGCAGGTCGGAGT GCGGCGCGCCGTCGACCTGCAGGTCG |
| NosTF*AleI*  NosTR*PacI* | N/A | CCATGACACCGCGGTGAGTAACATAGATGACAC CGCGGTTAATTAAGAATTTCCCCGATCGTTC |
| HKTQFwd  HKTQRev | At4g10310 | TGCAAACTGCGGATTTGTCC TGAGCAAAACCAAGAAGCAAGG |
| AVPQFwd  AVPQRev | At1g15690 | CCTCTCATTGTTGGTTTCTTC GATGTATTTTCTTGGCGTTGTC |
| GAPDHQFwd  GAPDHQRev | At3g26650 | TGGTTGATCTCGTTGTGCAGGTCTC GTCAGCCAAGTCAACAACTCTCTG |
| ACTINQFwd  ACTINQRev | At5g43500 | GAGTTCTTCACGCGATACCTCCA GACCACCTTTATTAACCCCATTTACCA |
| Trans AtHKT1;1Fwd  Trans AtHKT1;1 Rev | At4g10310 | TGAACGGCGTGTGGACATCA  CCCCCCCTCGAGTTAGGAAGA |
| Native AtHKT1;1Fwd  Native AtHKT1;1 Rev | At4g10310 | ATTAGTCACTTTTAGCATTC  ATTACAAGTTACAACAGGAA |
| HKT1Fwd  HKT1Rev | At4g10310 | ATGGACAGAGTGGTGGCAAA TTAGGAAGACGAGGGGTAAAGTATC |
| GaPDhFwd  GaPDh Rev | Os02g38920 | GGGCTGCTAGCTTCAACATC  TTGATTGCAGCCTTGATCTG |
| ActinFwd  Actin Rev | Os01g73310 | GAAGATCACTGCCTTGCTCC  CGATAACAGCTCCTCTTGGC |
| TubulinFwd  Tubulin Rev | Os01g59150 | TACCGTGCCCTTACTGTTCC  CGGTGGAATGTCACAGACAC |
| PpIaseFwd  PpIase Rev | Os03g10400 | AATCACTTCGCATCGGACAT  GCAAATCCTCGGCAGTAGAC |
| ElF1Fwd  ElF1 Rev | Os03g29260 | ATCTGGGAAATCATCGGTTCTG  AGATCGTCCACAATGGTCATCA |
| OsHKT1;5Fwd  OsHKT1;5 Rev | Os01g20160 | ACGACCCCATCAACTACAGCGTCC  TGCTCCACTTCCCTGAGAAGCCAAC |
| OsOVP1Fwd  OsOVP1 Rev | Os06g43660 | AGGCATCCTCTTCAAGTGG  GGACAGACAGTAATAACAAATAGG |
| OsOVP2Fwd  OsOVP2 Rev | Os06g08080 | TCACGAATAACGACTGCCAG  ACGCACCAGAGATGGGAG |
| OsOVP3Fwd  OsOVP3 Rev | Os05g06480 | CCATCGCTCAACATCCTCGTCAA  TGCTCAGCTTCATCAGAGGT |
| OsOVP4Fwd  OsOVP4 Rev | Os02g55890 | TTCGCCACGCACGGAGGA  CCACTACCACCAAAGCAACATT |
| OsOVP5aFwd  OsOVP5a Rev | Os02g09150 | CTCCTTGTTCACTGTGCCAC  TGAGTTGATGACTCCATTGTCTAC |
| OsOVP6aFwd  OsOVP6a Rev | Os01g23580 | GGTTCGGACGCTCACAA  ATACTCCGTGCGATTGTCAC |
| OsATPaseA1Fwd OsATPaseA1 Rev | Os06g45120 | GCTAATGCTGATGGACAA  CAGAAGGAAAATATGATGATTC |
| OsATPaseA2Fwd OsATPaseA2 Rev | Os02g07870 | TTCTGAGAGAAGATTATTTGGC  CCGTGCTTCGTCTTCTAG |
| OsATPaseCFwd  OsATPaseC Rev | Os01g42430 | GGTCTGTTTGGAGTGATTGTGG  AAGTCGCAGCAGTTATGTAGTC |
| OsHKT1;1Fwd  OsHKT1;1Rev | Os04g51820 | GAGCACTGTGGAGGAATTTTACCG  TAGTGAGTAGCCTACATTGCCGAAA |
| OsHKT1;3Fwd  OsHKT1;3Rev | Os02g07830 | GCTTACTTTGCCCTGATCTCCT  TGAATACCTCACCACCAATC |
| OsHKT1;4Fwd  OsHKT1;4Rev | Os04g51830 | GCGACTCTGGCAAACTGATA  GGTTCCTGTCTATGTGAAAATGAATA |
| OsHKT2;1Fwd  OsHKT2;1Rev | Os06g48810 | GTTAATTTTGTTGTTCTAGC  ATGAGGCTGGAAAGTGTCAG |
| OsHKT2;3Fwd  OsHKT2;3Rev | Os01g34850 | CTGCCATGAGAAGGCGTACAA  ATCGCATACTGATCGCTTCTGAT |
| OsHKT2;4Fwd  OsHKT2;4Rev | Os06g48800 | CTTGCCATGAGAAGCCATACAG  CTTGATTCTTGCATAACATCATCA |
| OsNHX1Fwd  OsNHX1Rev | Os07g47100 | GCTGATGACCAAAGGGAAGA  CCCGACGGCTCCAAATA |
