# Supplementary figures and images for: Improved Salinity Tolerance of Rice Through Cell Type-Specific Expression of AtHKT1;1
Source: PLoS One. 2010 Sep 3;5(9):e12571. doi: 10.1371/journal.pone.0012571 (PMC2933239; doi:10.1371/journal.pone.0012571)

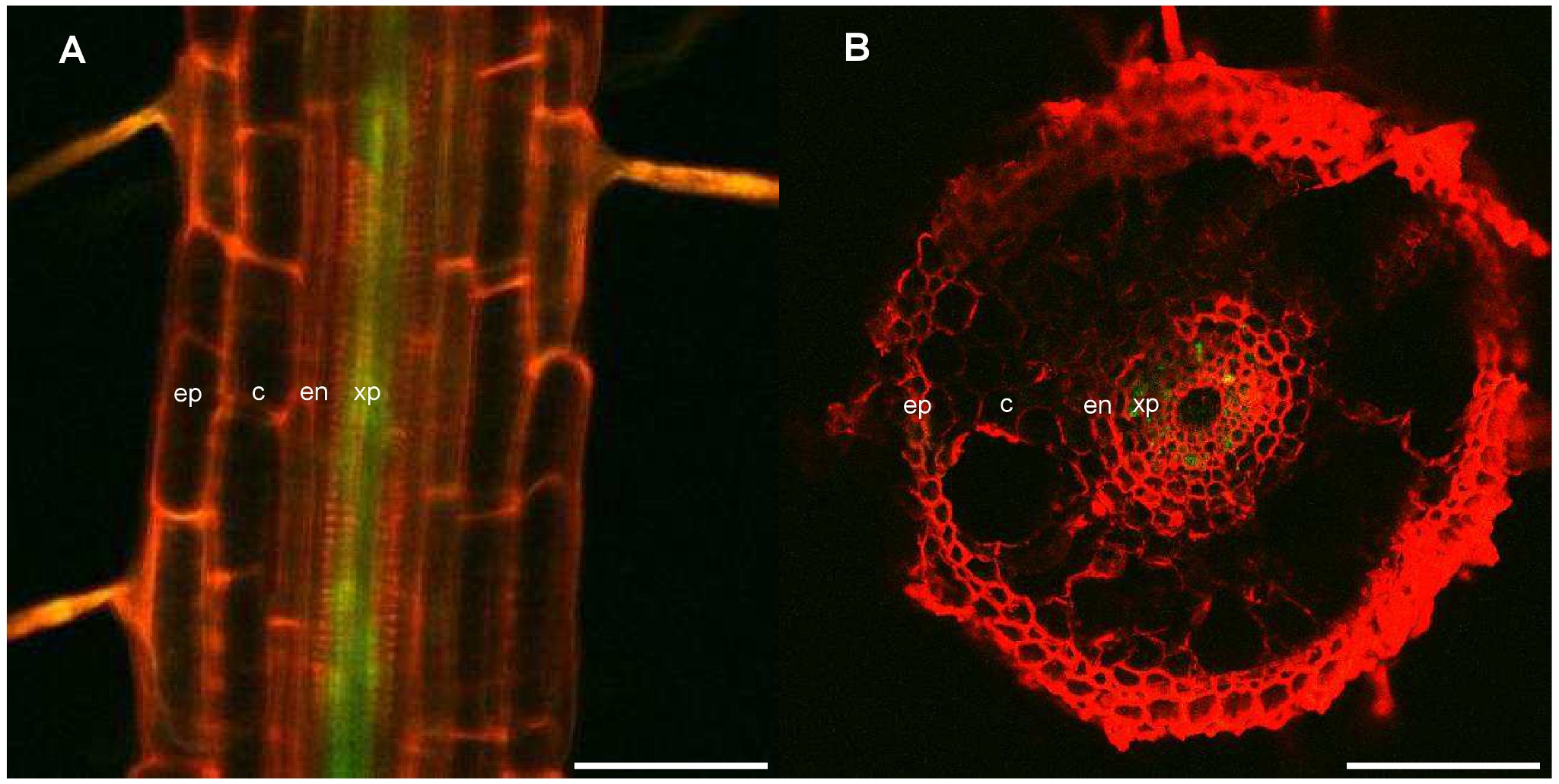

Supplement: Figure S1 — Images of the rice GAL4-GFP enhancer trap line ASG FO3. (A + B) Confocal laser microscope images of line ASG F03 showing GFP fluorescence specifically within the root xylem parenchyma [Scale bars = 100 µm in (A) and 100 µm in (B)]. Tissue was stained with propidium iodide. GFP and propidium iodide images were captured separately and overlaid to create the composite image. Tissues labelled include the epidermis (ep), cortex (c), endodermis (en), xylem parenchyma (xp) and metaxylem (mx). (2.65 MB TIF) [file pone.0012571.s005.tif]

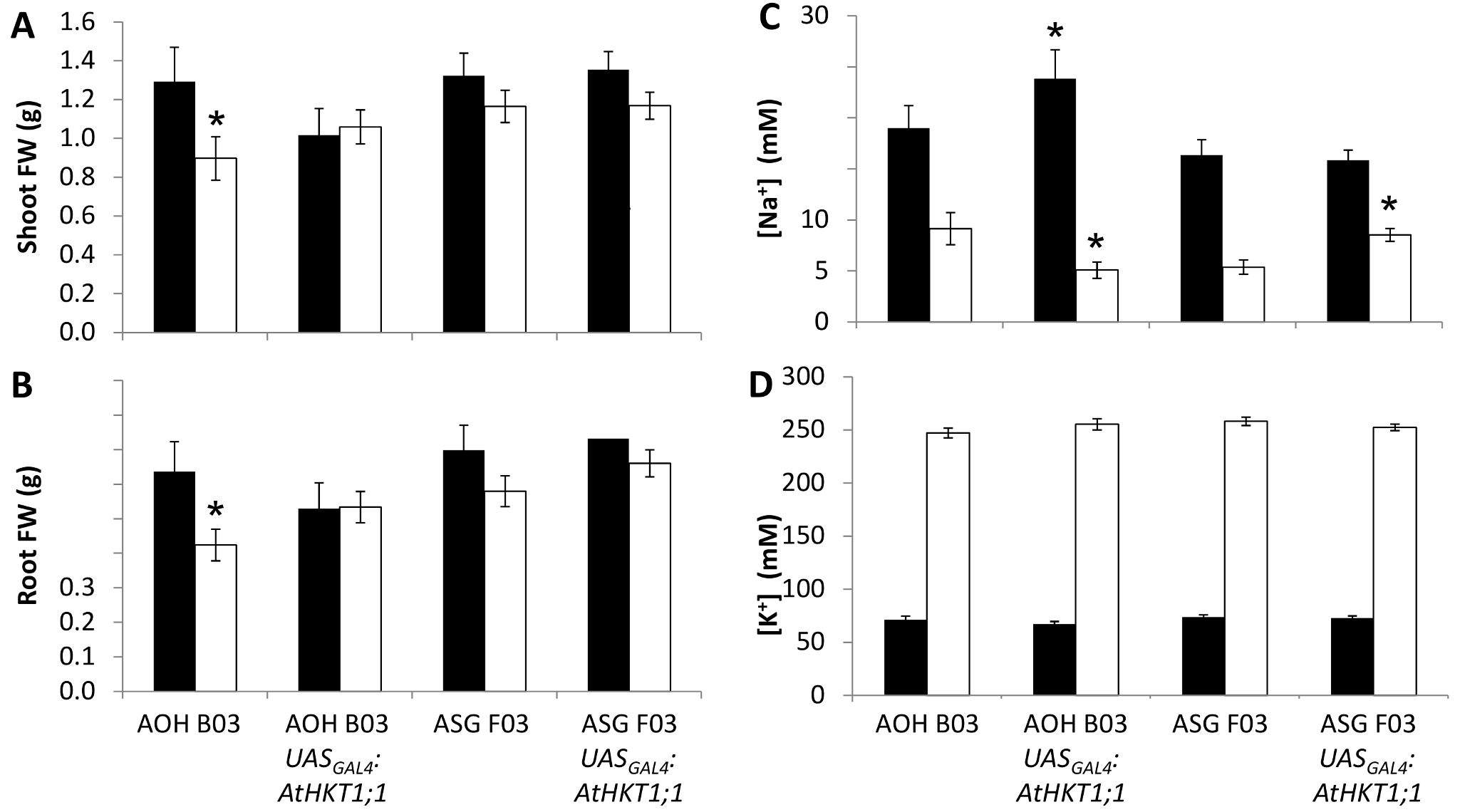

Supplement: Figure S2 — Fresh weight (FW) measurements and tissue Na+ and K+ concentration of T1 rice lines. Lines express AtHKT1;1 within the root cortical cells (AOH B03 UASGAL4:AtHKT1;1) or the root xylem parenchyma cells (ASG F03 UASGAL4:AtHKT1;1) and are compared with their respective background lines (AOH B03 and ASG F03). Measurements of FW of (A) shoots and (B) roots of control (black bars) and salinity stressed (open bars) rice plants grown for 5 d on 80 mM NaCl (n = 8, error bars represent SEM). Statistical significance between treatments for each line was determined via the Student's t-test, *P<0.05. Tissue concentration of (C) Na+ and (D) K+ in roots (black bars) and shoots (open bars) of rice plants grown for 5 d on 80 mM NaCl (n = 8, error bars represent SEM). Statistical significance from the respective control line for each tissue was determined via the Student's t-test, *P<0.05. (0.21 MB TIF) [file pone.0012571.s006.tif]

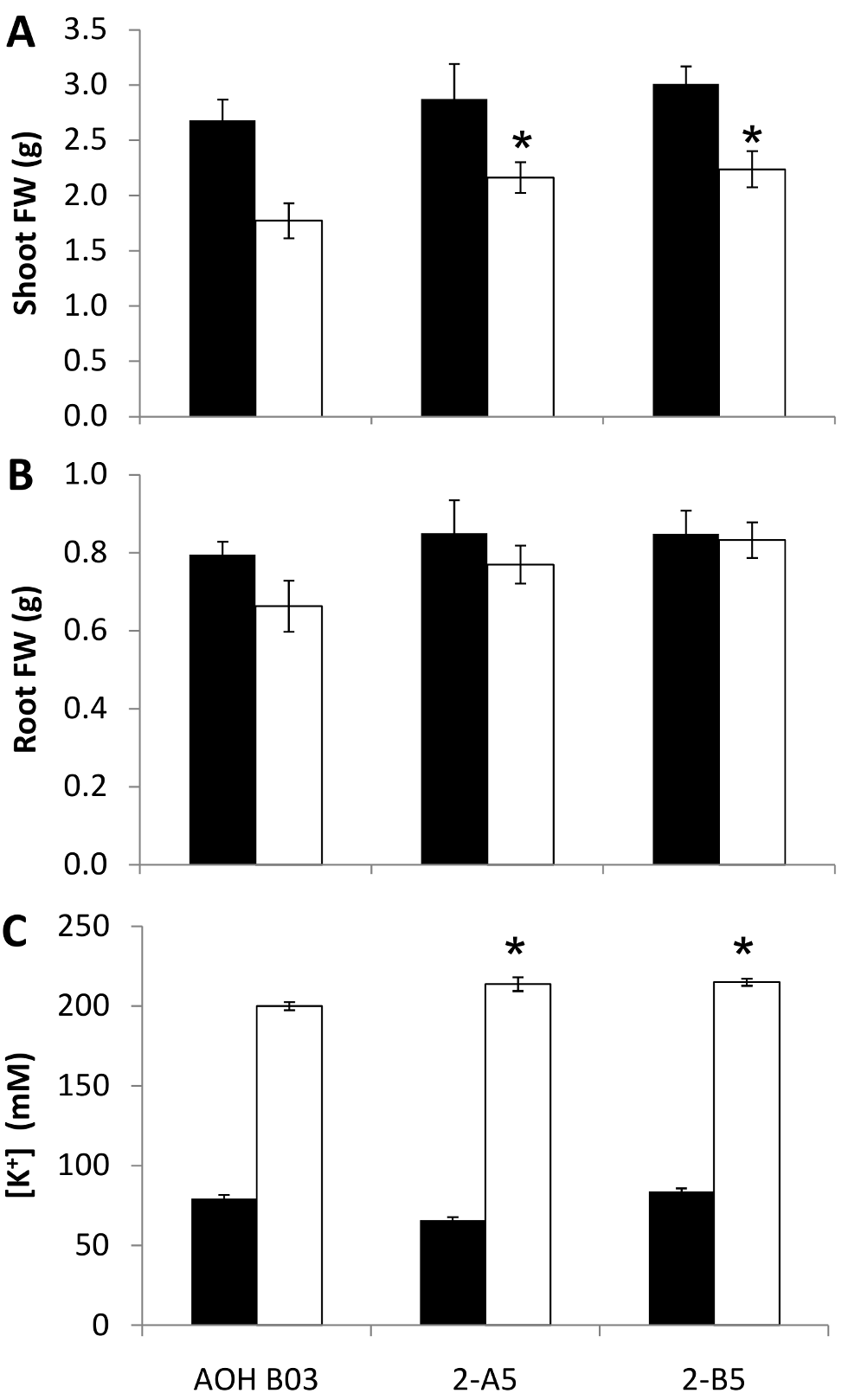

Supplement: Figure S3 — Fresh weight measurements of T2 rice plants. The FW was measured in (A) shoots and (B) roots of rice plants grown in control conditions (black bars) or in salinity stress conditions (open bars). (C) Concentration of K+ in the roots (black bars) and shoots (open bars) under salinity stress conditions. AOH B03 and two independent T2 AOH B03 UASGAL4:AtHKT1;1 lines (2-A5 and 2-B5) were grown for 5 d on nutrient solution containing 80 mM NaCl (n = 8, error bars represent SEM). Statistical significance from the AOH BO3 line was determined via the Student's t-test, *P<0.05. (0.12 MB TIF) [file pone.0012571.s007.tif]

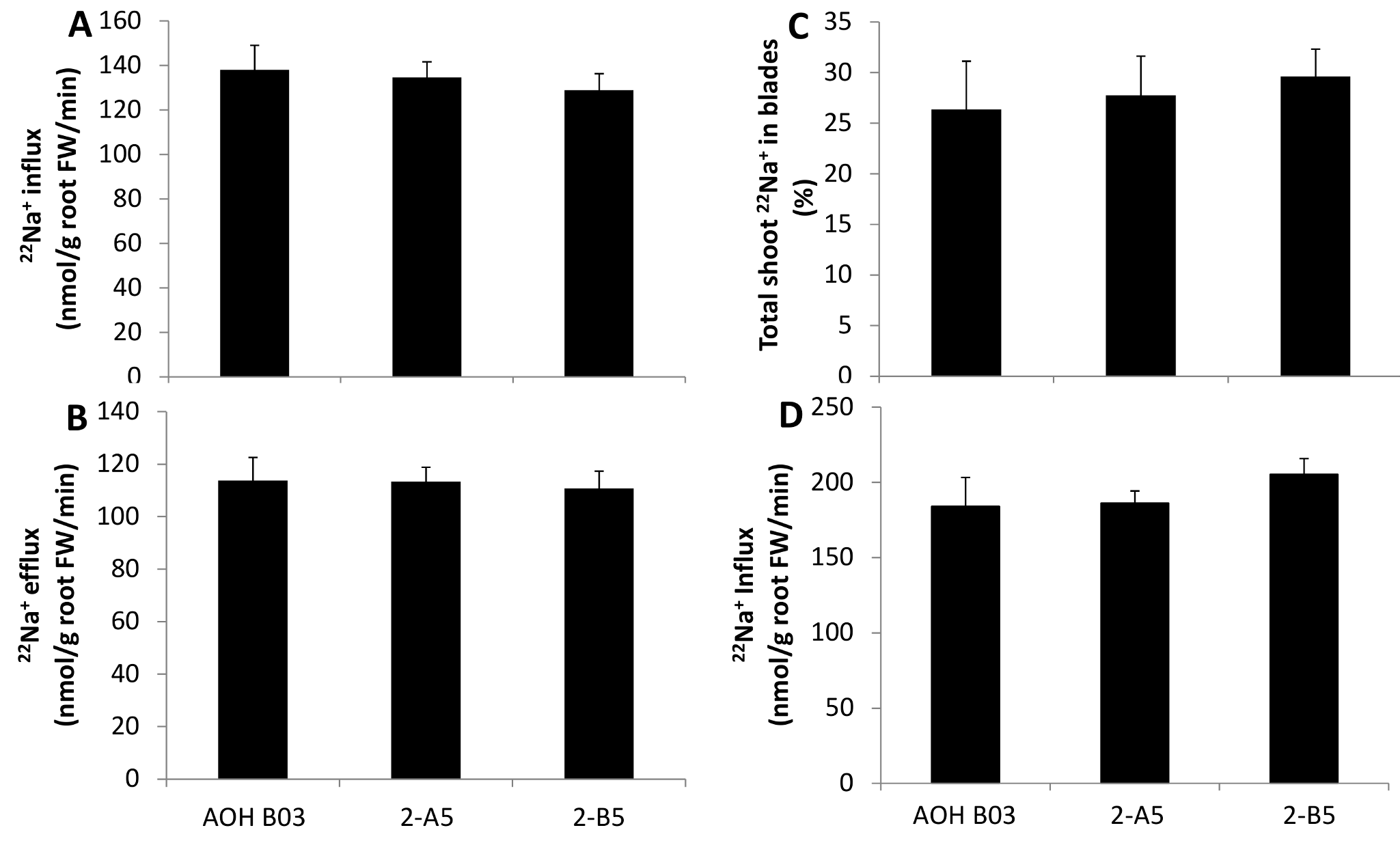

Supplement: Figure S4 — Flux measurements of Na+ inferred using the radiotracer 22Na+. Measurements were taken in AOH B03 and two independent T2 AOH B03 UASGAL4:AtHKT1;1 lines (2-A5 and 2-B5) grown for 3 d prior to experiment on nutrient solution containing 30 mM Na+ (n = 8, error bars represent SEM). (A) Measurement of the unidirectional influx of 22Na+ into non-excised rice roots exposed to an influx solution containing 30 mM Na+ for 2 min. (B) Root efflux of 22Na+ calculated by subtracting the net influx of 22Na+ into the plant (after 1 h exposure of the roots to a solution containing 30 mM Na+) from the unidirectional influx of 22Na+ (after a 2 min exposure of the roots to a solution containing 30 mM Na+). (C) Measurement of the sheath-to-blade transfer of 22Na+ expressed as a percentage of the total amount of 22Na+ in the shoot tissue that is contained within the leaf blades after exposing the roots of intact plants to a solution containing 30 mM Na+ for 1 h. (D) Measurement of the unidirectional influx of 22Na+ into rice roots excised immediately prior to experiment and exposed to an influx solution containing 30 mM Na+ for 2 min. (0.19 MB TIF) [file pone.0012571.s008.tif]

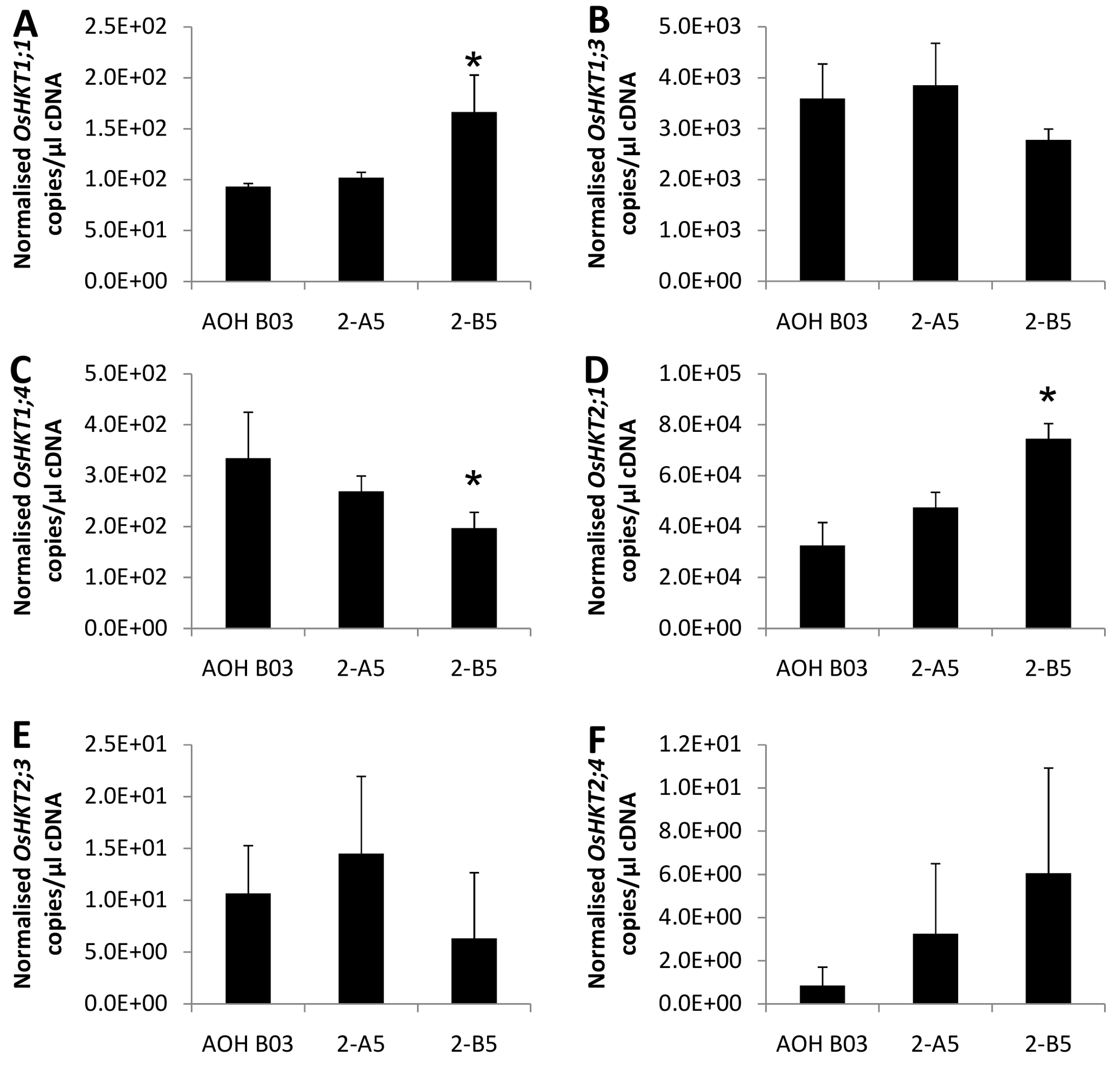

Supplement: Figure S5 — Expression of OsHKT family members measured using quantitative reverse transcriptase PCR. Included are AOH B03 and AOH B03 UASGAL4:AtHKT1;1 lines 2-A5 and 2-B5. Normalized expression of (A) OsHKT1;1, (B) OsHKT1;3, (C) OsHKT1;4, (D) OsHKT2;1, (E) OsHKT2;3 and (F) OsHKT2;4 in the roots of rice plants grown in 80 mM NaCl for 5 d. Each bar represents an average of four replicates (error bars represent SEM). Statistical significance from the AOH BO3 line was determined using Student's t-test, *P<0.05. (0.43 MB TIF) [file pone.0012571.s009.tif]

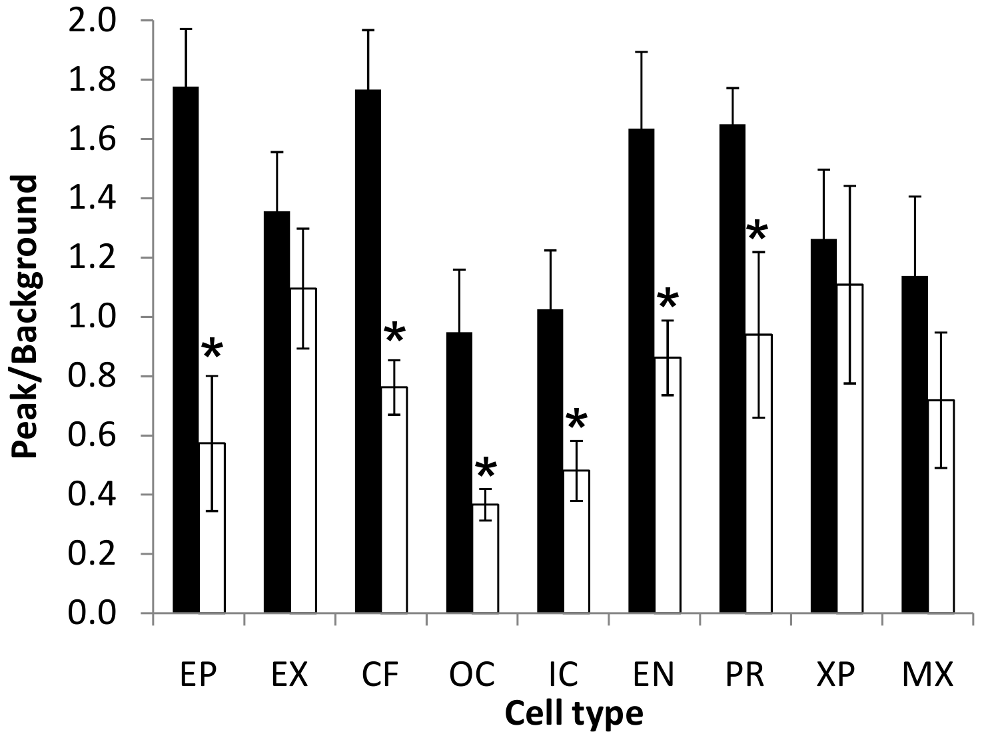

Supplement: Figure S6 — EDAX measurements of the amount of K+ (software measurement of peak/background) present in 9 root cell-types of rice. Lines include AOH B03 (black bars) and a AOH B03 UASGAL4:AtHKT1;1 line (2-B5) (open bars) and cell types are: EP - epidermis, EX - exodermis, CF - cortical fibres, OC - outer cortex, IC - inner cortex, EN - endodermis, PR - pericycle, XP - xylem parenchyma, MX - metaxylem. Each bar is an average of measurements made on three cells from each cell-type in the roots of three independent plants grown in a solution containing 50 mM Na+ for 5 d (error bars represent SEM). Statistical significance from the AOH BO3 line was determined via the Student's t-test, *P<0.05. (0.10 MB TIF) [file pone.0012571.s010.tif]

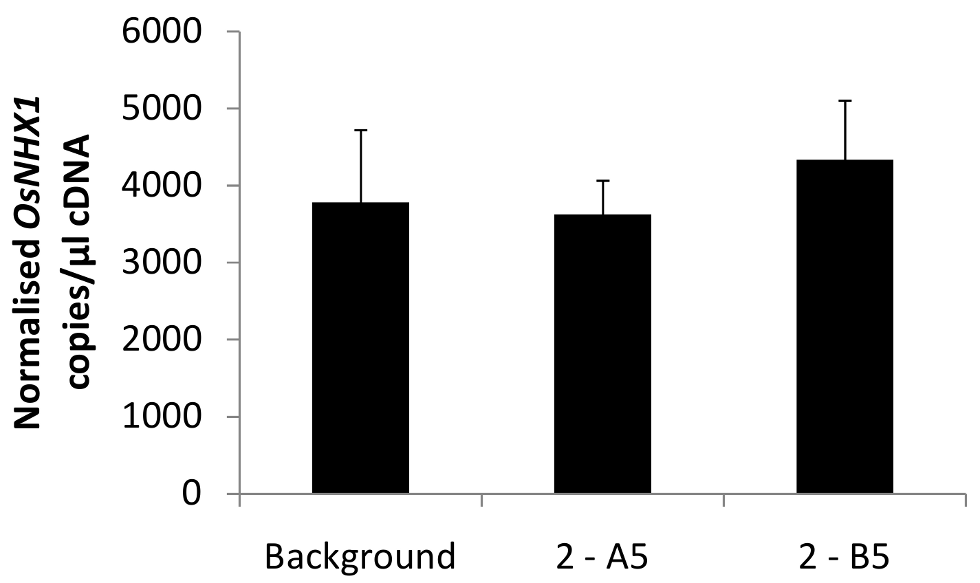

Supplement: Figure S7 — Expression of the Na+/H+ antiporter gene OsNHX1 measured using quantitative reverse transcriptase PCR. Included are AOH B03 and AOH B03 UASGAL4:AtHKT1;1 lines. Normalized expression of the vacuolar H+-pyrophosphatases (A) OsOVP1, (B) OsOVP2 and (C) OsOVP4 in the roots of rice plants grown in 80 mM NaCl for 5 d. Each bar represents an average of four replicates (error bars represent SEM). Statistical significance from the AOH BO3 line was determined using Student's t-test, *P<0.05. (0.05 MB TIF) [file pone.0012571.s011.tif]

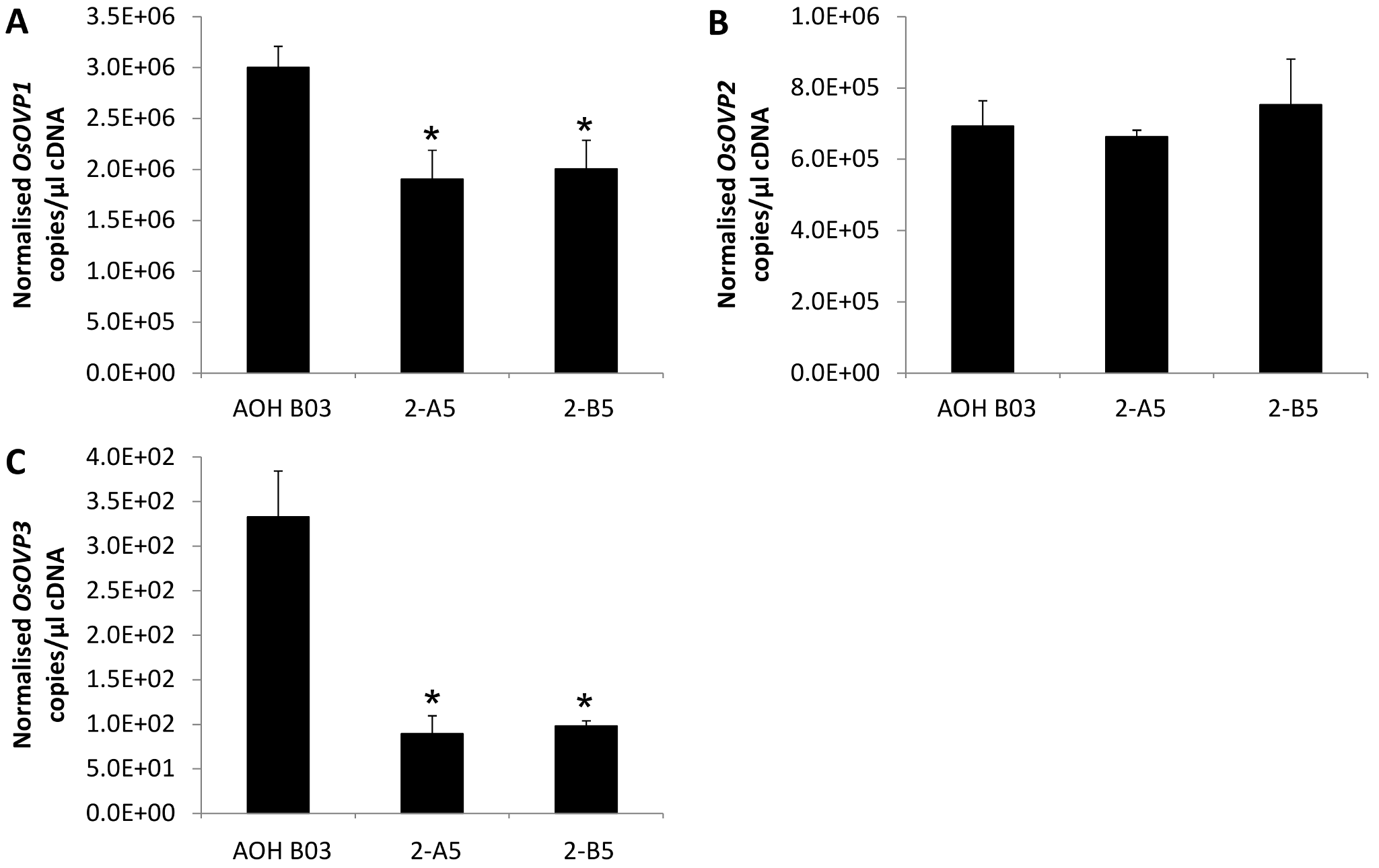

Supplement: Figure S8 — Expression of several rice vacuolar H+-pyrophosphatase genes measured via quantitative reverse transcriptase PCR. Included are AOH B03 and AOH B03 UASGAL4:AtHKT1;1 lines. Normalized expression of the vacuolar H+-pyrophosphatases (A) OsOVP1, (B) OsOVP2 and (C) OsOVP4 in the roots of rice plants grown in 80 mM solution for 5 d. Each bar represents an average of 4 replicates (error bars represent SEM). Statistical significance from the AOH BO3 line was determined via the Student's t-test, *P<0.05. (0.19 MB TIF) [file pone.0012571.s012.tif]
